# Sex chromosome aneuploidy impacts on human gene expression and regulation: a systematic review

**DOI:** 10.1186/s10020-025-01404-1

**Published:** 2025-12-30

**Authors:** Marcela Legue, Melanie Staszewski, Maya Mastronardo, Gisela Butera, Aleksandra Dakic, Armin Raznahan

**Affiliations:** 1 Section on Developmental Neurogenomics, Human Genetics Branch, National Institute of Mental Health, National Institutes of Health, Bethesda, MD USA; 2 Office of Research Services, NIH Library, National Institutes of Health, Bethesda, MD USA; 3 Department of Biology, Johns Hopkins University, Baltimore, MD USA; 4 Clinical Interventions and Diagnostics Branch, National Institute on Aging, National Institutes of Health, Rockville, MD USA

## Abstract

**Background:**

Sex chromosome aneuploidies (SCAs) are collectively common genetic disorders that impact diverse body systems. The molecular mechanisms by which an extra or missing sex chromosome increases clinical risk are not fully understood, but they likely involve imbalances in expression and regulation of dosage-sensitive genes. There has been a recent surge in transcriptomic and epigenomic studies on genomic effects of SCAs - making it an opportune time to: (i) map existing knowledge of SCA impacts on gene expression and regulation; (ii) resolve consensus findings on SCA dosage-sensitive genes; and (iii) define gaps and high priority areas for future research.

**Methods:**

Following the Preferred Reporting Items for Systematic Reviews and Meta-Analyses (PRISMA) guidelines (protocol registered on the international Prospective Register of Systematic Reviews PROSPERO CRD42024473984), we searched nine databases and screened the titles and abstracts of 2421 records, thoroughly evaluated 161 full-text articles, and identified 57 eligible studies for abstraction of methodological features and results.

**Results:**

Our review spans 18 years of research and encompasses samples from 930 individuals with SCAs and 2192 euploidic controls. The recent acceleration in publication rates outstrips that for biomedical research as a whole. Studies have only recently started to diversify away from the most studied SCAs (47,XXY and 45,X karyotypes), tissue types (blood-derived, gonadal) and measurement methods (transcriptomic analysis by RNAseq). We identify a core set of dosage-sensitive genes that are recurrently impacted by SCAs across multiple tissues. These genes concentrate in 3 protein-protein interaction networks that are predominantly enriched for chromatin remodelling, and represent candidate drivers of downstream phenotypes.

**Conclusions:**

This systematic review of SCA impacts on the human genome helps to target the future research efforts that are now needed to (i) address existing knowledge gaps by diversifying the karyotypes, tissues and genomic features analyzed, and (ii) test the causal role for recurrently dysregulated genes. Meeting these goals would provide a molecular foundation to drive both basic and clinical understanding of sex chromosome influences on human phenotypic variation.

**Supplementary Information:**

The online version contains supplementary material available at 10.1186/s10020-025-01404-1.

## Introduction

Sex chromosome aneuploidies (SCAs) are genetic conditions defined by the carriage of a sex chromosome count other than the typical euploid 46,XX or 46,XY karyotypes seen in females and males, respectively. These conditions are collectively common (~ 1/400 births) (Berglund et al. 2020; Nielsen and Wohlert 1991; Sánchez et al. 2023) and clinically relevant because of their potential to impact multiple organ systems. Klinefelter syndrome (KS or 47,XXY) is the most common SCA followed by other trisomies (47,XXX and 47,XYY), Turner syndrome (TS, including 45,X and mosaics) (Berglund et al. 2020; Nielsen and Wohlert 1991; Sánchez et al. 2023), and high grade aneuploidies (four or more sex chromosomes) (Kleczkowska et al. 1988; Spaziani et al. 2024; Linden et al. 1995). Diagnostic rates reflect frequency of early or severe manifestations rather than prevalence, being highest for TS, intermediate for KS (Berglund et al. 2020; Nielsen and Wohlert 1991; Sánchez et al. 2023), lowest for other trisomies (Berglund et al. 2019), and typically elevated for HGA due to their more severe phenotypes (Spaziani et al. 2024; Ricciardi et al. 2022).

Sex chromosome aneuploidies are associated with diverse clinical consequences across neurological, endocrine, cardiometabolic and musculoskeletal domains (Davis et al. 2016; Juul et al. 2023; Aly and Kruszka 2022) that all presumably stem from the effects of SCAs on genome function. Interestingly, sex chromosome dosage variations can have markedly diverse patterns of impact over different clinical traits. Some of SCA clinical phenotypes appear to track with sex chromosome dosage in a manner that is mirrored between SCAs involving chromosomal losses and gains. For example, individuals with 45,X manifest short stature, and height increases with additional chromosomes up to tetrasomic states (Hanson et al. 2024; Ottesen et al. 2010; Berry et al. 2025). Other clinical outcomes—such as increased risk for neurodevelopmental and psychiatric difficulties—are seen in both 45,X and supernumerary SCA groups (Sánchez et al. 2023; Whitman et al. 2021; Green et al. 2018; Bishop et al. 2011; Seidlitz et al. 2020; Levitis et al. 2024; Hall et al. 2025). Intriguingly, the specific profile of these neuropsychiatric risks shows subtle but significant differences between different SCA karyotypes such as: differences in the relative vulnerability of visuospatial vs. verbal tasks with loss vs. gain of sex chromosomes (van Rijn and Swaab 2011; Hong and Reiss 2014; Fezza et al. 2024; Fales et al. 2003), and differential severity of mood vs. social problems with gains of X- vs. Y-chromosomes (Ross et al. 2009; Rau et al. 2021; Schaffer et al. 2024; Berry et al. 2024). There are also phenotypes that emerge in a context-dependent fashion. For example, presence of an additional X-chromosome induces much more severe and clinically overt hypogonadism in males than females (Bonomi et al. 2017; Davis et al. 2020).

The diverse patterns of karyotype-phenotype mapping in SCAs underline the need to better understand the molecular mechanisms by which atypical X and Y-chromosome dosage impacts genome function - including detection of recurrently implicated dosage-sensitive genes that are likely to drive downstream phenotypic outcomes. This understanding is not just important for revealing mechanistic processes that could potentially be measured or manipulated for clinical benefit in SCAs, but may also cast light on how sex chromosome dosage shapes normative sex differences between 46,XX and 46,XY individuals (Arnold 2020, 2012; Raznahan and Disteche 2021; Mankiw et al. 2017). These many clinical and basic science motivations for better understanding SCA impacts on genome function are amplified by three recent trends: the rapid growth of prenatally detected SCA cases by non-invasive prenatal testing (Gadsbøll et al. 2024; Lau et al. 1982); the growing recognition that many individuals with SCAs still go undiagnosed (Sánchez et al. 2023; Davis et al. 2024; Viuff et al. 2015); and the rapid expansion in our technological capacity for analysis of genome structure and function (Brittain et al. 2017; Satam et al. 2023). Collectively, these considerations make it a good time to systematically review existing knowledge to date and plan next steps for the field (Tallaksen 2023; Oliveira et al. 2024).

Here, we present the first Systematic Review [PRISMA guidelines (Page et al. 2020), PROSPERO CRD42024473984] of studies examining the genome-wide impacts of X- and/or Y-chromosome dosage variation in humans. We sought to: (i) map existing knowledge of SCA impacts on gene expression and regulation; (ii) resolve consensus findings on SCA dosage-sensitive genes; and (iii) define gaps and high priority areas for future research. Our review incorporates studies that use unbiased genome-wide measures of transcriptomic (e.g. microarrays, next generation sequencing [NGS], etc.) or epigenomic (e.g. methylation arrays or sequencing, chromatin accessibility and conformation, etc.) changes in SCA samples. We first examine the content of all available studies meeting our selection criteria, considering rates of publication over time and distribution of studies across different karyotypes, tissues and genomic assays. Then, by aggregating comparable reports regarding SCA impact on transcriptomic and epigenetic properties of the genome, we reveal a growing consensus on those highly dosage sensitive genes that are recurrently impacted by SCAs across multiple tissues. Finally, we highlight outstanding gaps in the available literature to help prioritize key questions and associated study designs for future research.

## Results

### Study selection process

**Fig. 1:**
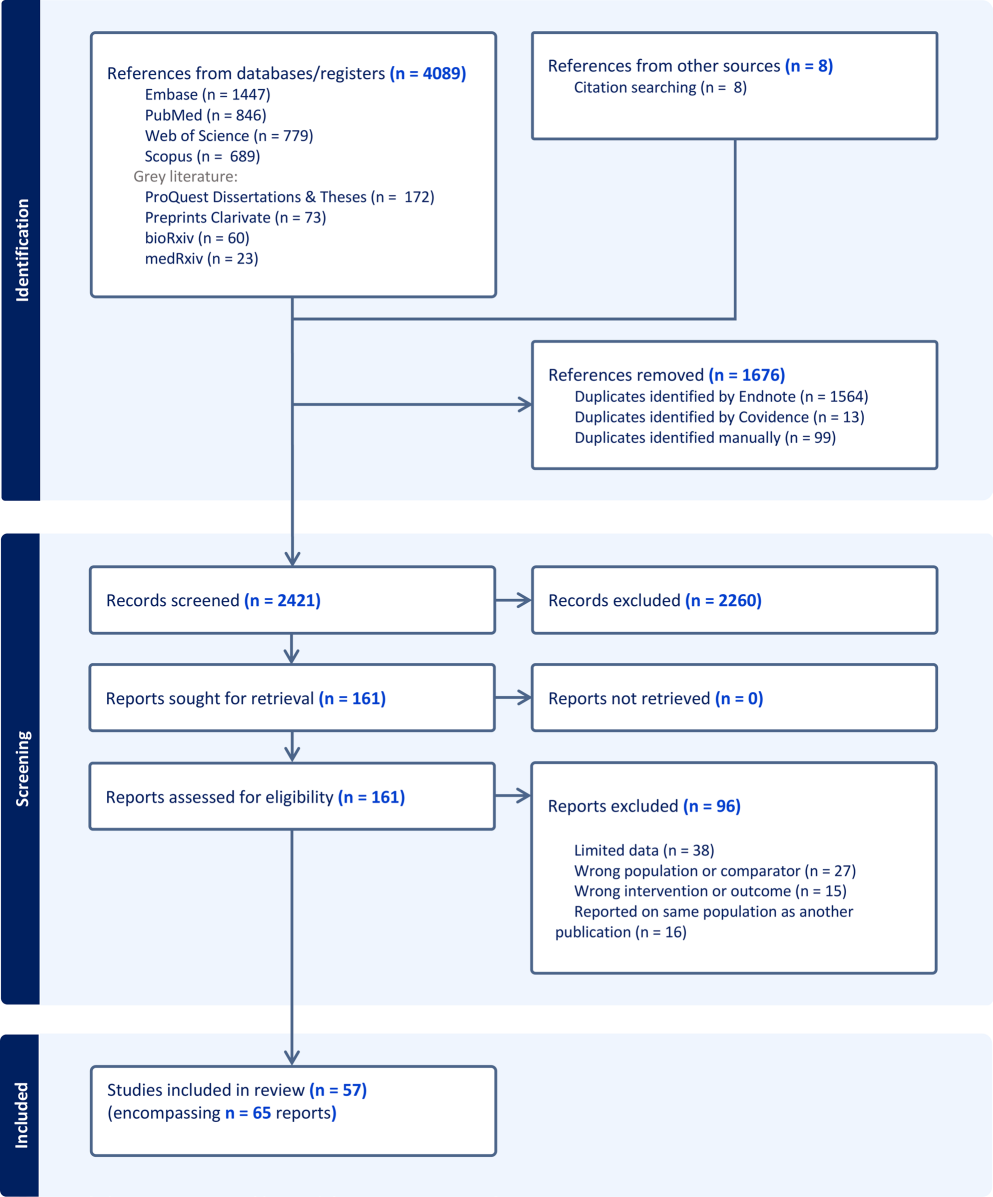
PRISMA flowchart of the study selection process. Flow diagram of steps for selecting eligible studies. Steps included identification and screening of records (title and/or abstracts), full-text assessment of reports (articles or publications), and final selection of studies (investigation on a defined group of participants with one or more intervention and outcomes). Since a study may have multiple reports, the number of final number studies is less than the reports included

The selection process was developed under the PRISMA guidelines (Fig. 1) in collaboration with a trained librarian, and designed to cast an intentionally wide net to ensure comprehensive coverage of the target literature on the transcriptomic and epigenetic impacts of sex chromosome aneuploidy (SCA). We searched for original articles, dissertations, theses, or reports on Embase, Pubmed, Web of Science, Scopus, ClinicalTrials.gov, Clarivate ProQuest, Clarivate Preprint Citation Index, bioRxiv and medRxiv, without any filter from database inception to February 2025 (see full search strategy in Methods and File S1, and Fig. S1). This search was then further expanded using the references and citations of all articles identified by this initial database review. Our search protocol retrieved 4089 total records (title/abstracts), with 2421 remaining after removal of duplicates. Two independent reviewers screened and filtered these 2421 titles and abstracts using Covidence (Covidence 2024), and then carefully evaluated the resulting 161 full-text reports (publications) for eligibility criteria. We applied carefully selected inclusion criteria to ensure a minimum standard for meaningful comparison, and optimize the trade-off between internal and external validity. The studied population included human individuals with SCAs - excluding instances of partial, mosaic or somatic SCA. The techniques included were assays or methods designed for unbiased genome wide measurement of gene expression or epigenomic regulatory mechanisms such as DNA methylation or chromatin structure, and we excluded studies that solely evaluated DNA sequence, or that were focused on a predefined group of genes. Controls included human participants with reported non-mosaic euploid karyotype or a different non-mosaic SCA. The full detail of inclusion and exclusion criteria is in Table S1. Application of these inclusion and exclusion criteria identified 65 eligible reports, from which we defined a final set of 57 studies for downstream data extraction and analysis once individual publications derived from the same study cohort (as defined by Page et al. 2020) were merged into a single entity (study), as summarized in Table *S2* (Cimino et al. 2017; Salemi et al. 2019, 2021, 2018 merged into Cimino et al. 2017; D’Aurora et al. 2017, 2015 merged into D’Aurora et al. 2017; Viuff et al. 2023; Johannsen et al. 2022; Just et al. 2025 merged into Viuff et al. 2023; San Roman et al. 2023, 2024; Blanton et al. 2024 merged into San Roman et al. 2023).

### Trends in SCA studies and population characteristics

The 57 SCA studies included in this Systematic Review encompassed a total of 4682 samples across 3122 participants (930 aneuploid cases and 2192 euploid controls), 27 different tissue types and 11 different techniques for genomic measurements. The number of samples exceeded the number of participants because some studies analyzed multiple tissues or performed multiple assays on the same individual, and do not reflect technical replicates. Table 1 summarizes the key characteristics for each individual study included in this systematic review (full details in Table S2). Included studies originated from a total of 20 different countries and used samples ranging in life-stage from blastocysts to octogenarians. The adopted designs (Dey et al. 2020) could be classified as matched case control (20 studies, total sample size 610, SCA sample size range 1–67); unmatched case-control (25 studies, total sample size 35, SCA range 1–35); cross-sectional (9 studies, total sample size 1908, SCAs size range1–132) or case reports (3 studies, total sample size 3, SCA range 1–1). The risk of bias for individual studies was assessed with the ROBINS-E tool (Higgins et al. 2024), and the most frequently flagged criteria were small sample size or not-matched design, mainly affecting the ‘confounding factors’ and ‘selection of participants’ bias domains. Fig. S2 summarizes the risk assessment by domain, and File S2 details objective criteria and specific operationalization by study.

**Table 1.**
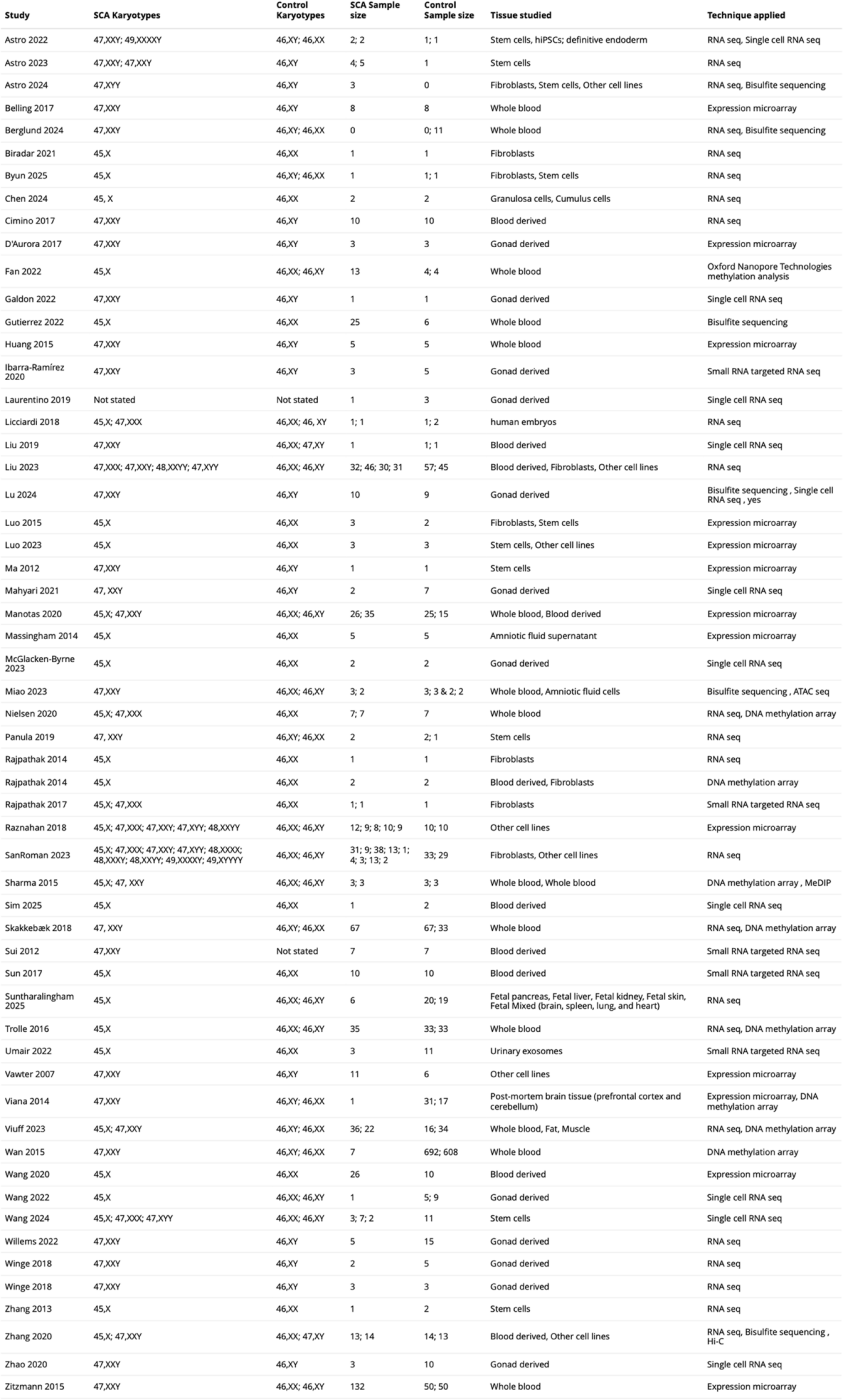
Characteristics of selected studies. Summary of included studies on transcriptomics and epigenomics in sex chromosome aneuploidies. Aneuploidies included, sample sizes, tissues studies and techniques applied are shown for individual studies (Viuff et al. 2023; San Roman et al. 2023; Astro 2024; Berglund et al. 2024; Chen et al. 2024; Lu et al. 2024; Wang et al. 2024, 2022, 2020; Byun et al. 2025; Sim et al. 2025; Suntharalingham et al. 2025; Wan et al. 2015; Belling et al. 2017; Miao et al. 2023; McGlacken-Byrne 2023; Luo et al. 2023; Astro et al. 2023, 2022; Willems et al. 2022; Umair et al. 2022; Gutierrez et al. 2022; Galdon et al. 2022; Fan et al. 2022; Biradar et al. 2021; Zhao et al. 2020; Zhang et al. 2020, 2013; Nielsen et al. 2020; Ibarra-Ramírez et al. 2020; Panula et al. 2019; Liu et al. 2019, 2023; Laurentino et al. 2019; Winge et al. 2018, 2018; Skakkebæk et al. 2018; Licciardi et al. 2018; Huang et al. 2015; Viana et al. 2014; Rajpathak et al. 2014; Rajpathak and Deobagkar 2014; Massingham et al. 2014; Sui et al. 2012; Ma et al. 2012; Mahyari et al. 2021; Manotas et al. 2020; Raznahan et al. 2018). For other demographic and outcome measures see Table S2.

**Fig. 2:**
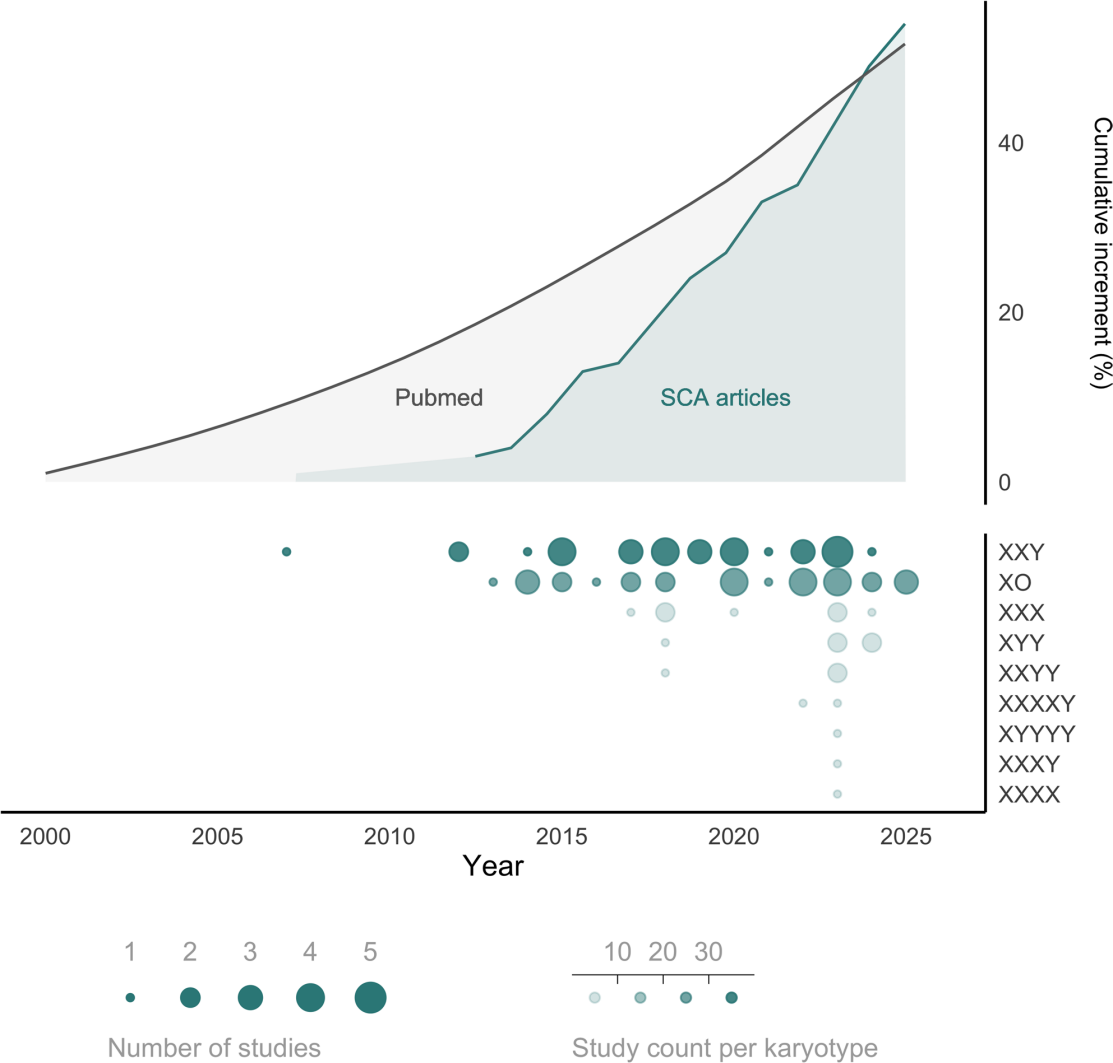
Timeline for cumulative frequency of SCA studies and included karyotypes. Cumulative increment of studies across time (top). Temporal distribution of SCA karyotypes studied (bottom). Note that 2025 publications only represent the first two months of the year due to our review’s selection process cutoff date (February 26th, 2025)

The articles identified by our Systematic Review span 18 years of genomic research on SCAs, with the earliest eligible study published in 2007 (Vawter et al. 2007). The accrual rate of SCA publications rose sharply after 2012 (Fig.2 - top panel) increasing at a qualitatively faster rate than the accrual of biomedical publications as a whole (as proxied by the cumulative rate of all Pubmed indexed scientific publications), and paralleling the broader acceleration of genomic studies during the post-2010 genomic era. Annotating SCA studies over time by the karyotypes included (Fig. 2 - bottom panel) showed that Turner 45,X and Klinefelter 47,XXY syndromes are the longest studied karyotypes, followed by less frequent trisomies (47,XXX and 47,XYY), with studies on the rarer high grade aneuploidies (48,XXXY, 48,XXXX, 49,XXXXY and 49,XYYYY) only becoming available after 2022.

**Fig. 3:**
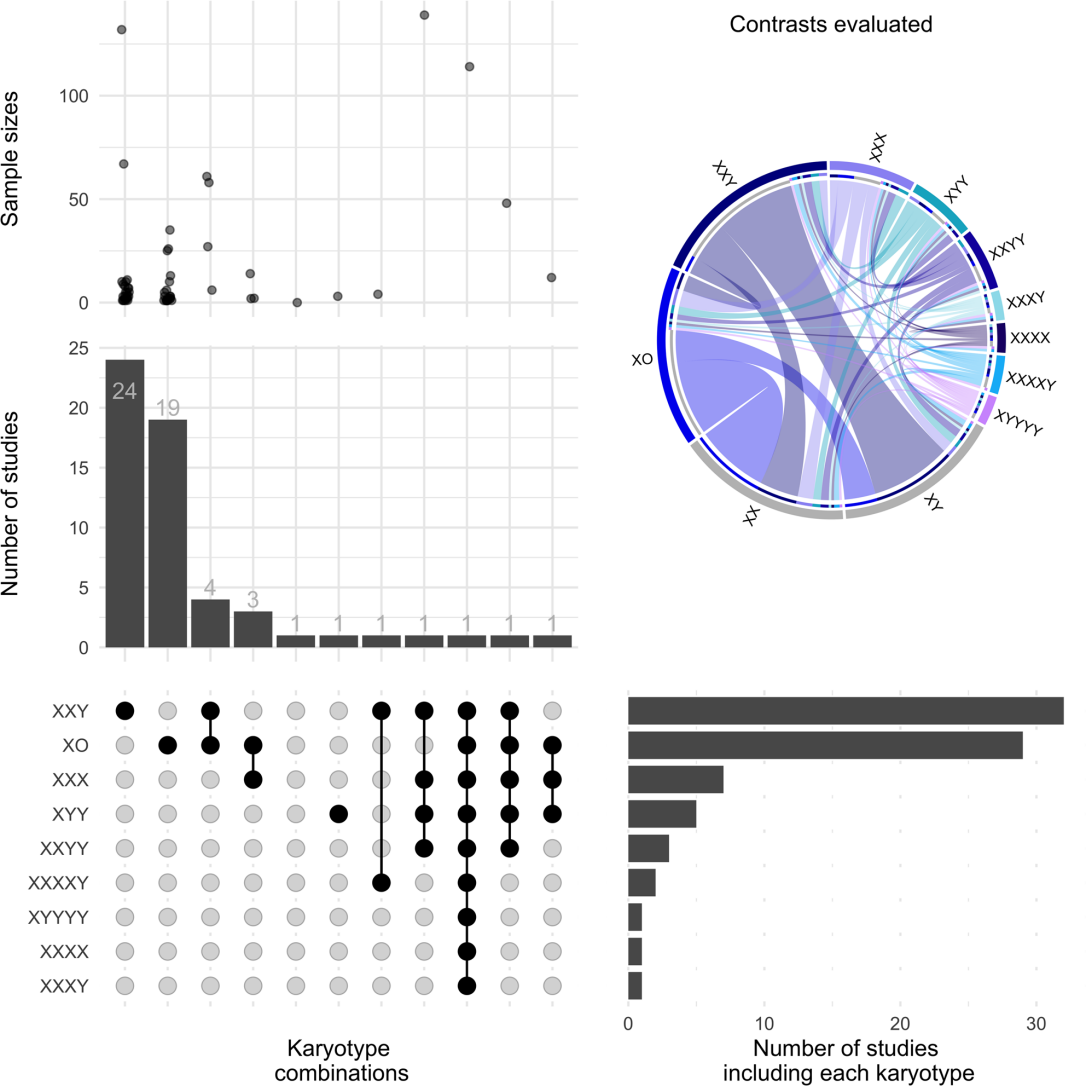
Types and frequency of sex chromosome aneuploidies included in different studies. Combination of sex chromosome aneuploidies by study (bottom-left), number of studies on each combination (center) and sample size of aneuploid cases (top-left). Number of studies including each karyotype (bottom-right). The prevalence of observed pairwise comparisons between karyotypes is shown as a circus plot (top-right) with thicker lines indicating more prevalent contrasts

Examining the diversity of studied karyotypes revealed a strong focus on particular SCA karyotypes and group contrasts. The majority of studies (44/57, 77%) considered a single SCA karyotype, which was typically 47,XXY or 45,X (55% and 43% of single-karyotype studies, respectively), with only a minority of studies including 2 or more karyotypes (2 karyotypes: 8/57, 14% studies; ≥3 karyotypes: 4/57, 7% studies) (Fig. 3, bottom left). A breakdown of all 930 SCA participants by karyotype echoed these trends—the majority of them (*n* = 741, 80%) had either 47,XXY trisomy or 45,X monosomy. A much smaller fraction of cases represented the remaining two sex chromosome trisomies (13% either 47,XYY or 47,XXX) and only 7% of samples displayed all other possible SCA karyotypes (Fig. 3, top left). As shown in Fig. 3 (top-right), a small subset of all possible pairwise karyotype group contrasts dominated the literature, with ~ 33% of all contrasts either involving comparisons between 45,X and 46,XX (16%), or 47,XXY and 46,XY (17%). Furthermore, 35% of contrast involved inter-aneuploidy comparisons.

### SCA samples, assays and genomic elements studied

**Fig. 4:**
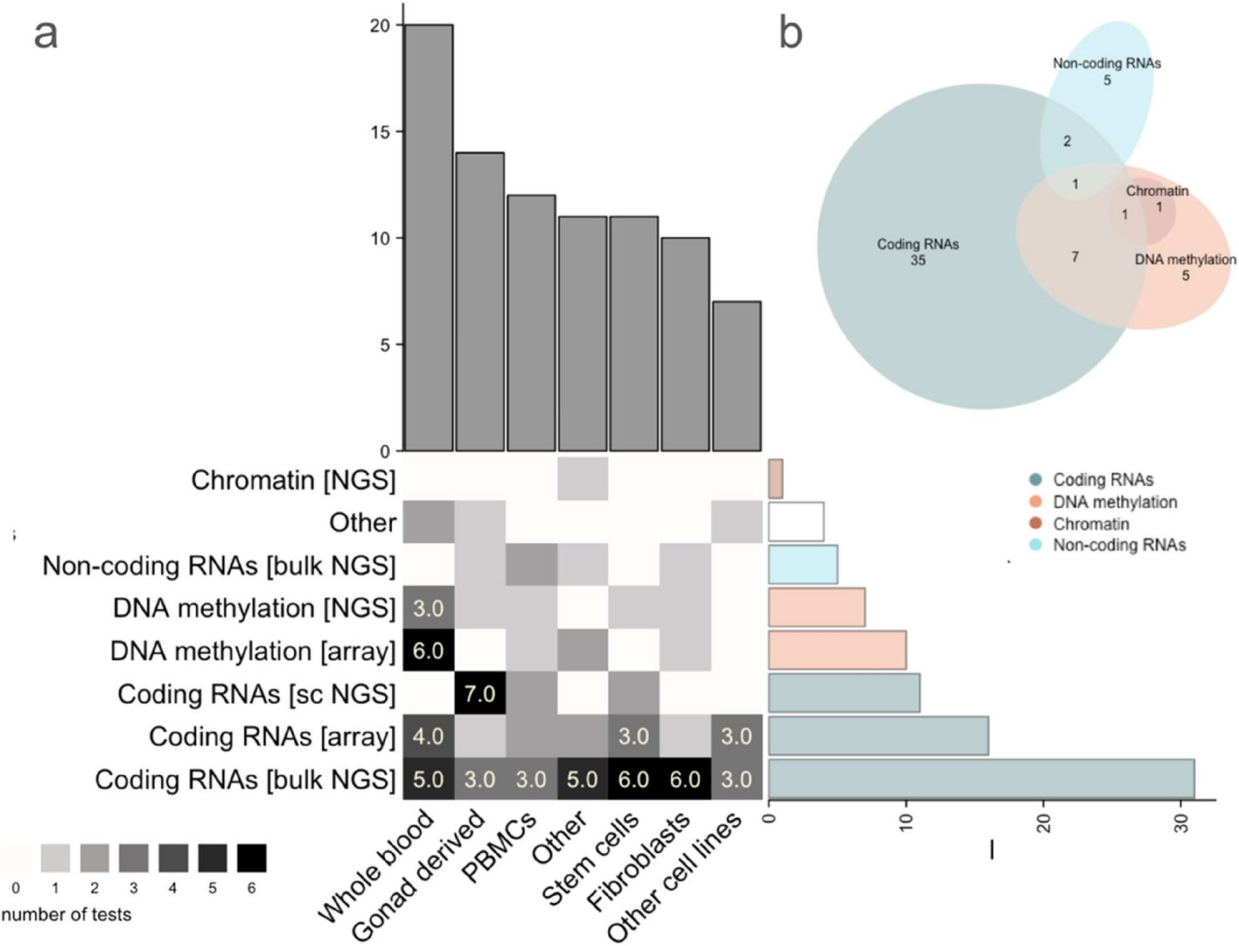
Sample types, techniques and genomic components measured. Sample types and assays from included studies. **a** Sample types, techniques and genomic components targeted. Repeated uses of a technique or sample type within a study are each counted as separate uses. **b** Venn Diagram depicting overlapping of different genomic levels by each study

Just as available studies were concentrated on certain karyotypes (Fig. 3), so too were they concentrated on certain tissue and cell types (Fig. 4). The included biosamples represented a variety of sources—such as cell lines, biopsies, postmortem tissue, prenatal embryonic, and amniotic fluid cells. The most recurrently examined tissue types by far were blood-derived, gonadal samples, and stem cells. Blood-derived samples (whole blood, peripheral blood mononuclear cells (PBMC) and lymphoblastoid cell lines (LCLs)) accounted for approximately 68% of studies, while gonadal tissues represented 25% (i.e. collectively 93%). Stem cells were predominantly human induced pluripotent stem cells (hiPSCs) and human embryonic stem cells (hESCs)] (Fig. 4). Thirteen studies evaluated more than one tissue, and only eight of them performed inter-tissue comparisons within the same individuals. Notably, despite the association of all SCA karyotypes with increased risk for neuropsychiatric difficulties, only one available study examined primary neural tissue [post-mortem prefrontal cortex and cerebellum] transcriptome and methylome, from one 47,XXY individual with schizophrenia (Viana et al. 2014). In addition, two recent studies utilized in vitro iPSC-derived neural cell lines—specifically neural stem cells from 47,XYY individuals (*n*= 3; Astro, 2024) and neuronal cells from 47,XXY; 47,XYY; 47,XXX; and 48,XXYY cases (*n*= 8; Liu, 2023).

The possible analytic approaches of the included studies, were organized under the following categories: (1) the phenotype class being considered (i.e. molecular phenotype, such as transcriptome or epigenome); (2) the specific genomic component being measured (coding RNA, non-coding RNA, DNA methylation, chromatin structure); (3) the measurement technology being applied (e.g. microarrays, NGS, etc.). Across the 57 included studies, the 4 aforementioned genomic components were evaluated through 10 different technologies [NGS-based methylation tests, bulk RNA-seq, single-cell RNA-seq, ATAC-seq, scTrio-seq, Hi-C, Stereo-seq, and microarray-based expression and methylation tests]. Some studies implemented more than one technology for evaluating more than one genomic feature over more than one tissue or cell type. As a result, the 57 studies together provided 85 separate tests for genomic impacts of SCAs (File S2, Fig. S3).

The vast majority of all 57 studies (*n* = 51; 90%) included gene expression measurements. The most utilized technology for gene expression assessment was bulk RNA-seq (*n* = 36, 42% of tests), followed by expression microarray (*n* = 16; 19%) and single-cell RNA-seq (*n* = 11, 13%). Most of the bulk RNA-seq and microarray tests (*n* = 31 and *n* = 15, respectively) and all the single-cell RNA-seq tests, focused on expression of coding genes, making this the most studied genomic feature in SCAs (*n*= 57; 67% of tests). Five RNA-seq tests were exclusively focused on non-coding genes (Umair et al. 2022; Ibarra-Ramírez et al. 2020; Sun et al. 2017; Rajpathak et al. 2017; Sui et al. 2012) and in three instances the analysis was performed together with coding genes (Cimino et al. 2017; Luo et al. 2023; Viuff et al.^2023^). The non-coding transcripts evaluated included miRNAs (Cimino et al. 2017; Umair, 2022; Ibarra-Ramírez et al. 2020; Sun et al. 2017; Rajpathak, 2017; Sui et al. 2012) lncRNAs (Cimino et al. 2017; Winge et al. 2018; Rajpathak et al. 2014; Luo et al. 2023) and circRNAs (Luo et al. 2023).

Measurement of SCA epigenetic impact was predominantly completed using array-based DNA methylation tests (*n*= 11), carried out mainly on whole blood (Viuff et al. 2023; Berglund et al. 2024; Wan et al. 2015; Nielsen et al. 2020; Skakkebæk et al. 2018; Trolle et al. 2016; Sharma et al. 2015). Other sample sources evaluated using this technique were PBMCs (Rajpathak et al. 2014), fat and muscle (Viuff et al. 2023), and brain (Viana et al. 2014). The 6 tests using NGS tools for analysis of methylation encompassed a range of applications including targeted methylation sequencing (Zhang et al. 2020; Gutierrez et al. 2022), reduced representation bisulfite sequencing (RRBS) (Astro 2024) whole-genome bisulfite sequencing (WGBS) (Miao et al. 2023) and single-cell whole-genome bisulfite sequencing (as part of scTrio-seq) (Lu et al. 2024). Epigenetic features other than DNA methylation were much less studied in SCAs with only two studies investigating alternative features. Miao et al. (2023) measured extra X chromosome impacts on transcriptionally active open chromatin by using ATAC-seq on 47,XXY amniotic fluid cells (alongside measures of blood DNA methylation profiled by bisulfite sequencing); and Zhang et al. (2020) studied 45,X and 47,XXY effects on chromatin interactions with Hi-C in LCLs and integrated their findings with transcriptomics and bisulfite sequencing.

Combined consideration of genomic technology and sample type in SCA research revealed the most common combinations to be bulk RNA-seq analysis of stem cells and fibroblasts (*n* = 6 tests each), single-cell RNA-seq assays of gonadal tissue (*n* = 7 tests), and methylation-array assays of whole blood (*n* = 6 tests) (Fig. 4). Both technology and tissue shows uneven utilization across years and their evolving trends are depicted in Fig. S4.

In summary, we observed a strong focus of research on a subset of all available methods for genome-wide analysis, with NGS measurement of the transcriptome being the most utilized assay.

### Genes consistently sensitive to sex chromosome dosage across tissues

The studies within our systematic review included 8 cohorts (Viuff et al. 2023; Astro 2024; Suntharalingham et al. 2025; Astro et al. 2023; Willems et al. 2022; Zhang et al. 2020; Winge et al. 2018; Liu et al. 2023) in which SCA effects on expression of coding genes were assessed using RNA-seq on at least 3 biological replicates (the minimum recommended for analysis of differential expression (Zhang et al. 2014). These studies collectively provided 49 lists of significantly differentially expressed genes (DEGs) through comparative analyses including at least one SCA group. In contrast, no other modality of genome measurement provided more than 6 of such lists.

We harnessed the opportunity offered by those 49 RNA-seq differential expression analyses identified by our Systematic Review to aggregate information across multiple studies and rank sex chromosome and autosomal genes by the proportion of published group contrasts in which they were reported as a DEG (henceforth “DEG frequency”)—a putative proxy for SCA dosage sensitivity. Given the sparsity of available studies, this process necessarily required combining DEG lists across different tissues. For sex-chromosome genes, this combination of information across different tissues is supported by the fact that cis-effects of SCAs on gene expression are known to be highly consistent across tissues (Liu et al. 2023). For autosomal genes, this combination has the potential to recover the generic trans-effects of aneuploidy that have been reported across multiple aneuploidies and sample types (Sheltzer et al. 2012; Mendioroz et al. 2015; Hervé et al. 2016)—even though these trans-effects appear to be more tissue specific than cis-effects (Makarevitch and Harris 2010; Kahlem et al. 2004; Liu et al. 2023; Blanton et al. 2024).

**Fig. 5:**
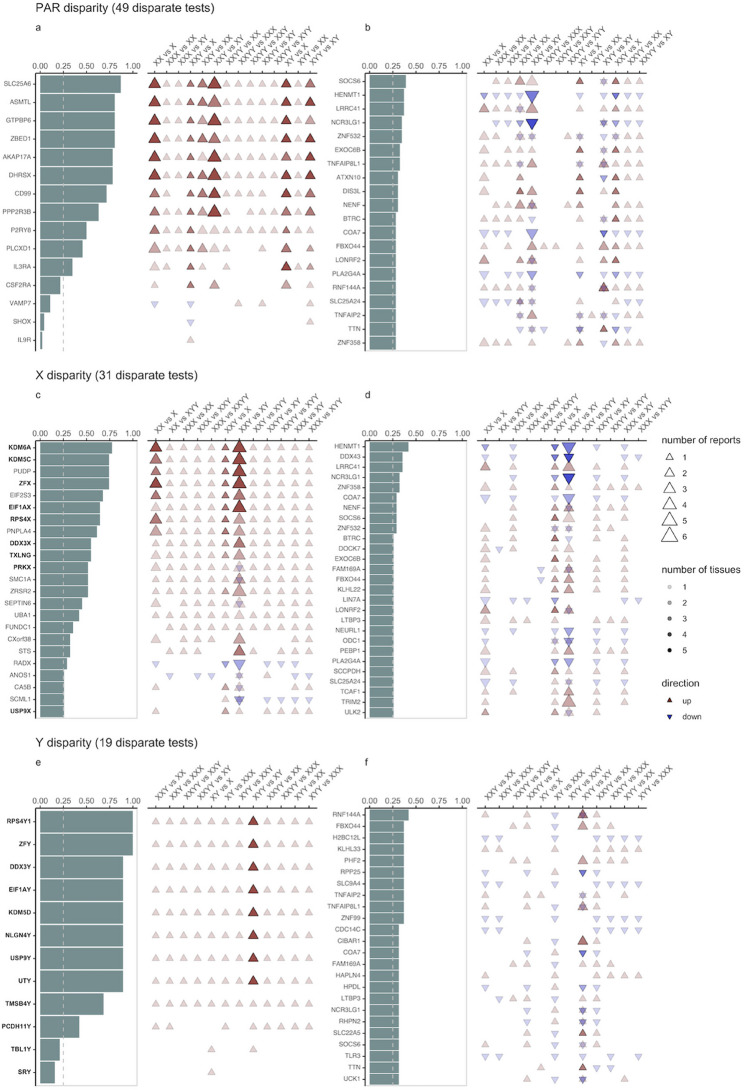
Recurrently reported DEGs in gene expression studies of SCAs. Left column bar plots rank genes by the proportion of contrasts in which they are reported DEGs (dashed vertical gray line denoting proportion of 25%). Right column dot plots show the direction of differential expression for each gene in the informative contrasts (arrow direction and color), the number of reports of differential expression (arrow size) and the number of tissues in which differential expression has been reported (arrow color saturation). Gametolog genes are in bold. Blood-derived and fetal tissues have been collapsed for visualization, itemized information is available in Table S3** a**,** b**. Recurrent PAR and autosomal DEGs for PAR-disparate contrasts. **c**,** d.** Recurrent X-linked and autosomal DEGs for X-disparate contrasts. **e**,** f.** Recurrent Y-linked and autosomal DEGs for Y-disparate contrasts

Of the 49 DEG lists included in this aggregation analysis, 31 contrast reports involved a disparity in X-chromosome number (reflecting a collective sample size of 261 aneuploid individuals and 237 controls in the originating studies), 19 involved disparity in Y-chromosome number (sample size of 206 aneuploidy individuals and 193 controls) and 46 involved a disparity in pseudoautosomal region (PAR) dosage (collectively involving 264 aneuploid samples and 237 controls). We collected the union of all unique DEGs for each of these specific contrasts, and ranked them by their DEG frequency across the respective dosage-altered sex chromosome region (Fig. 5). This process was done separately on autosomes. Higher ranked genes are those that are most consistently reported as DEGs given dosage disparity of a particular sex chromosome region (PAR, X-chromosome or Y-chromosome).

For the 46 PAR-disparate contrasts, the list of unique DEGs included 18 PAR genes (49% of all PAR coding genes) and 14,433 autosomal genes (78% of all autosomal genes), (Fig. 5, Fig S5, Table S3). We observed a clear relative enrichment of PAR versus autosomal genes at higher DEG frequency thresholds (e.g. for a DEG frequency of ≥ 25%, overrepresentation OR = 206.3, Fisher exact test p-value = 1.027357e-20). There were eight genes with a DEG frequency of >50%, all of which were PAR genes. The top ranked of these was *SLC25A6* (87%, encoder of a mitochondrial translocase). All the PAR genes with a DEG frequency ≥ 25% were consistently upregulated with increasing PAR copy number across reported contrasts and tissues, that included blood-derived, fat, fetal-derived, fibroblasts, iPSCs, muscle, and neural stem cells (Fig. 5a, Fig S5, Table S3). The 3 highest ranking autosomal genes by DEG frequency for PAR-disparate contrasts (all with DEG frequencies >35%)—were *SOCS6 *(a suppressor of cytokine signaling that promotes mitochondrial fission [Lin et al. 2013]); *LRRC41* (a nuclear protein involved in the ubiquitination pathway [Schenková et al., 2012]); and *HENMT1*(a methyltransferase involved in piRNA stabilization [Lim et al. 2015]).

For the 31 X-disparate contrasts, the list of unique DEGs included 417 X-linked coding genes (51% of all X-linked coding genes) and 11,313 autosomal genes (61% of all autosomal genes) (Fig. 5, Fig.S5, Table S3). At greater cut-offs of DEG frequency, the overrepresentation of X-linked chromosome genes became more pronounced (e.g. for a DEG frequency ≥ 25%, OR = 19.75, Fisher exact test p value = 7.421128e-19). All 13 genes with DEG frequencies >50% were X-linked, and 8 of these were X-linked gametologs (*KDM6A*, *KDM5C*, *ZFX*, *EIF1AX*, *RPS4X*, *TXLNG*, *DDX3X*, *PRKX*). The gametolog genes with the highest DEG frequencies were the chromatin remodelers *KDM6A* (77%, a H3K27me3 demethylase), *KDM5C* (74%, a H3K4me3 and H3K4me2 demethylase) and the transcriptional activator *ZFX* (74%, a zinc finger protein). Non-gametolog X-linked genes with high DEG frequencies included *PUDP* (74%, a pseudouridine 5’-phosphatase) and *EIF2S3 *(68%, a translation initiation factor involved in the recruitment of methionyl-tRNA_i_ to the 40(S) ribosomal subunit)—which have both been reported to escape X inactivation (Yen et al. 1992; Balaton et al. 2015). Most of the X-linked genes with a DEG frequency >25% displayed a consistent pattern of upregulation with increasing X chromosome copy number, across blood-derived, adipose, fetal-derived, and muscle tissues. This pattern of ubiquitous upregulation had some exceptions. For example genes such as *PRKX*,*SMC1A*,* SEPTIN6*, exhibited downregulation in iPSCs. Interestingly, the X-linked gene *RADX*, a DNA binding protein relevant for genomic stability, was downregulated in all reported contrasts, which were derived from blood or fat (Fig. 5a, Fig S5, Table S3). As expected, autosomal genes had lower DEG frequencies than X-linked genes across X-disparate contrasts. For example, 27 autosomal genes had DEG frequencies above 25% (0.1% of all autosomal genes) as compared to 23 X-linked genes (2.9% of all X-linked genes). The 3 autosomal genes with the highest DEG frequencies across X-chromosome disparate contrasts were *DDX43 *(36%, DDX43 an helicase essential for chromatin remodeling [Tan et al. 2023]), *HENMT1* (42%), and *LRRC41* (36%).

For the 19 Y-disparate contrasts, the list of unique DEGs included 12 Y-linked coding genes (27% of all Y-linked coding genes) and 10,658 unique autosomal genes (57% of all autosomal genes) (Fig. 5, Table S3). All 9 genes with DEG frequencies >50% were Y-linked, and the top ranked of these*—*both with DEG frequencies of 100%*—*were *ZFY* (a transcriptional regulator functionally linked with its gametolog *ZFX*) and *RPS4Y1* (a ribonucleoprotein that is a structural component of the 40 S subunit). All the Y-linked genes with a DEG frequency ≥25% were consistently upregulated with increasing Y-chromosome copy number. These Y-disparate contrasts represented blood-derived, fetal-derived, fibroblasts, iPSCs and neural stem cells (Figure 5a, Fig SX, Table S3). Sixty-eight autosomal genes were reported as differentially expressed in more than 25% of Y-disparate contrasts. The highest ranking autosomal gene by DEG frequency was *RNF144A* (40%, a ring finger protein that promotes degradation through ubiquitination), with the next set of highest ranking autosomal genes all having DEG frequencies of 36%. This gene set included *FBXO44 *(a silencer of repetitive elements [Shen et al. 2021])​​, *RPP25 *(a ribonuclease [Huang et al. 2010]) and other genes listed in Fig.5.

While there is a high proportion of autosomal genes identified as DEGs at least in one tissue and contrasts (78% in PAR-disparate contrasts), these reported DEGs show lower convergence than sex chromosome cis-effects (most autosomal DEGs appear in only 1–2 studies/tissues, and the maximum autosomal DEG proportion in any single contrast is 42%, well below the highest sex chromosome proportions of 100%). This phenomenon likely reflects biological variability across tissues and contrasts rather than higher impact on the autosomes. This finding is consistent with the tissue-specificity of autosomal trans-effects reported previously in the literature (Liu et al. 2023; Makarevitch and Harris 2010; Kahlem et al. 2004; Liu et al. 2023; Blanton et al. 2024).

### Functional relevance of most reported genes by genomic compartment

Given prior evidence that SCA effects on gene expression are nested within coordinated networks of genes that interact within shared functional pathways (Zhang et al. 2020; Liu et al. 2023), we sought to explore how recurrent SCA protein-coding DEGs relate to genome biology by integrating them within a protein-protein interaction (PPI) network. As input for these analyses, we considered coding genes that had >25% DEG frequencies for PAR-, X-, or Y-disparate contrasts. This cut-off balanced network density and gene set size (see Methods), yielding a set of 137 sex chromosome and autosomal genes for downstream PPI analyses (using the String v.12 database [Aleksander et al. 2023]), Methods). As expected, this DEG list was highly enriched for sex chromosome as compared to autosomal-encoded genes (*n* = 44 and 93, respectively; overrepresentation OR = 11.2, chi^2^ test p-value < 2.2e-16).

**Fig. 6:**
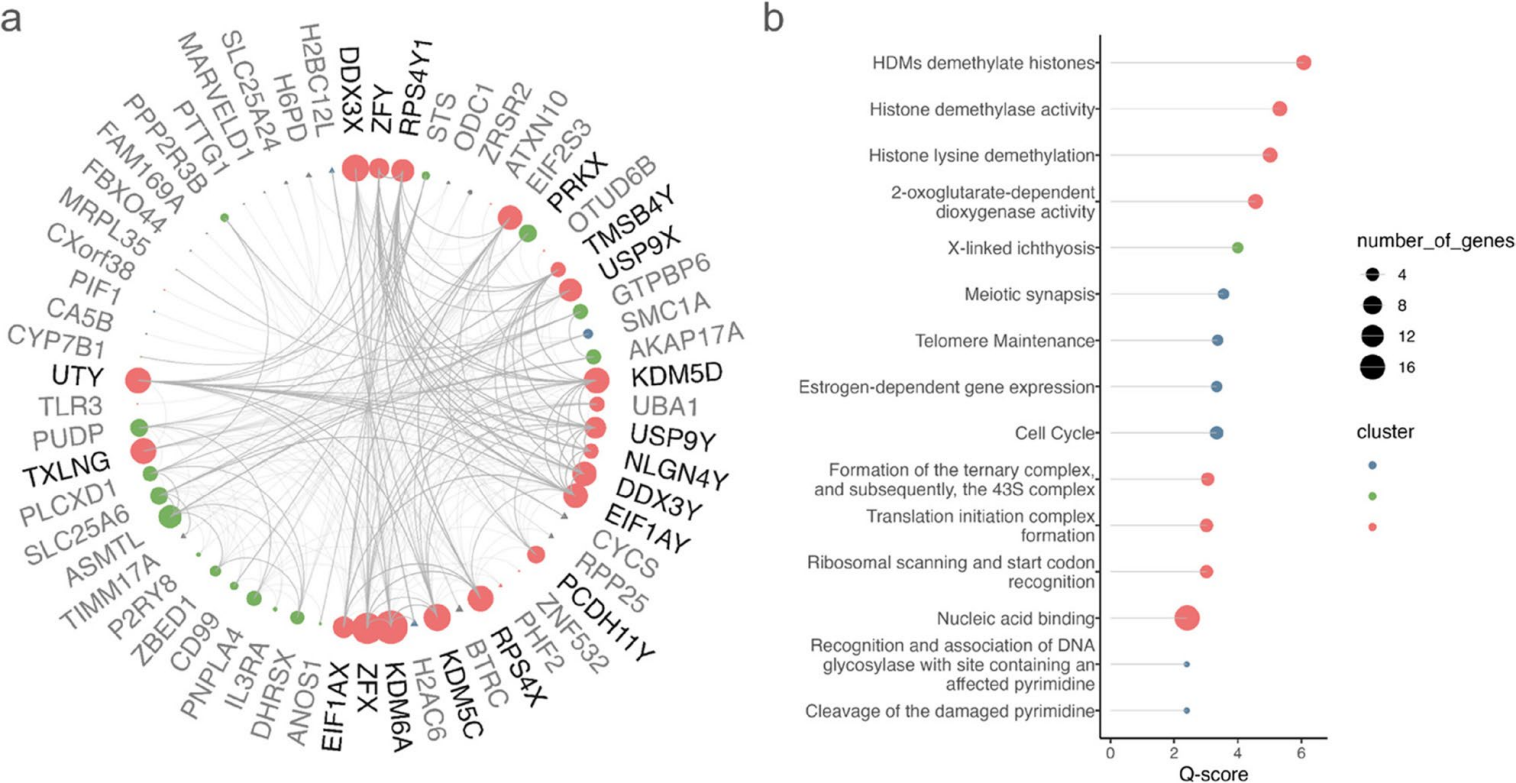
A protein-protein interaction network based on the proteins encoded by the most recurrently differentially expressed genes in SCAs.** a**, Largest connected component comprising 81% of the nodes of the network of genes reported in more than 25% of contrasts. Node size represents node degree. Sex chromosome-encoded are represented as circles, autosomally encoded as triangles. Sex chromosome gametologs are in bold. **b**, Top 15 functional annotations for the three main clusters, ranked by enrichment scores. Color represents cluster membership and size depicts number of genes. Full list with gene names, p-values and scores in Table S4

The pairwise binary PPI adjacency matrix was constructed for the proteins encoded by the 137 DEGs in our input dataset. This network exhibited an edge density of 0.2% (an edge taken to be present at confidence scores above the default cut of 400, Methods). This edge density was significantly elevated relative to the distribution of edge densities for 10,000 randomly drawn gene sets of similar size (21-fold, P_perm_ =9.999e-05), and marginally elevated when random gene sets were drawn with the added constraint of degree matching (1.4-fold, P_perm_<0.07). Seventy-five of the 137 input proteins had one or more edges in the PPI network, and 81% of these fell into one large connected component which possessed at least 3 distinguishable communities (clusters) based on the Walktrap algorithm (Pons 2005) (Fig.6 and 3 main communities colored: red, green, blue). We conducted an enrichment analysis for the proteins of these clusters using selected database annotations provided by String (Cellular Component, Biological Process, and Molecular Function from Gene Ontology [Ashburner et al. 2000; Aleksander et al. 2023], KEGG [Kanehisa et al. 2025; Kanehisa 2019; Kanehisa and Goto 2000], DISEASES [Pletscher-Frankild et al. 2015; Grissa et al. 2022], COMPARTMENTS [Binder et al. 2014], Reactome [Milacic et al. 2024], Pfam [Mistry et al. 2021], and WikiPathways [Pico et al. 2008]).

The largest PPI cluster of recurrent SCA DEGs (red) included 26 proteins, with high overrepresentation of those encoded by sex chromosome genes (*n* = 19, enrichment OR = 61.7, chi-squared test p-value < 2.2e-16), and KDM6A, KDM5C, and ZFX as their 3 highest-degree nodes. The proteins within this cluster were enriched for annotations involving histone modifications, translational initiation and nucleic acid binding processes. The second largest PPI cluster (green) included 18 proteins which were also enriched for those encoded by genes on sex chromosomes (*n* = 16, enrichment OR = 172.3, p-value < 2.2e-16), and had ASMTL, PRKX, SCL25A6 and PUDP as their highest degree nodes. The main enrichment for this cluster was X-linked ichthyosis. The smaller PPI cluster (blue), included among its proteins the X-linked gene SMC1A, and two autosomal H2 clustered histones, H2AC6 and H2BC12L—and was enriched for annotations related to meiotic synapsis and telomere maintenance, estrogen-dependent gene expression and cell cycle. These three PPI clusters were strongly interconnected (Fig. S6), with highest degree inter-cluster nodes being KDM6A (red cluster), PRKX (green) and SMC1A (blue). Most edges connected red and green clusters, mainly between KDM6A, ZFX, RPS4X (red) and PRKX, ASMTL, and PUDP (green). All the red and blue cluster connections were through KDM6A, KDM5C and UTY to SMC1A, H2AC6 and H2BC12L. These nodes form PPI bridges between clusters—potentially supporting the coordination of SCA impacts across different molecular pathways.

## Discussion

Our study provides the first Systematic Review on the rapidly expanding field of SCA impacts on human gene expression and regulation. This field of research is critical for understanding how atypical sex chromosome dosage drives clinical risk in SCAs, and also nominates dosage sensitive genes that may shape sex chromosome dosage influences on normative sex differences between 46,XX and 46,XY groups. By comprehensively mapping existing knowledge, we identify gaps to be prioritized in future work and resolve recurrently implicated gene sets.

We find that the bulk of our knowledge regarding SCA impacts in the human genome stems from studies of gene expression differences in 47,XXY and 45,X samples compared to euploidic controls, based on blood-derived and gonadal tissues. This emphasis on frequently diagnosed syndromes, available techniques and accessible tissues is logical, and has potential to uncover important mechanistic insights as detailed below, but also leaves many crucial questions regarding the proximal transcriptomic consequences of SCAs that may drive downstream phenotypes.

First, there is a dearth of knowledge regarding the transcriptomic effects of several clinically impactful SCAs other than 45,X and 47,XXY; including the sex chromosome trisomies 47,XYY and 47,XXX, and the rare but most clinically severe high-grade SCAs such as 48,XXYY, 48,XXXY and 49,XXXXY. Relatedly, we lack studies comparing multiple SCAs, which are necessary to understand the molecular basis for shared and distinct clinical outcomes across karyotypes. Such studies have only just started to emerge in recent years (Viuff et al. 2023; San Roman et al. 2023; Astro 2024; Astro et al. 2022; Zhang et al. 2020; Nielsen et al. 2020; Liu et al. 2023; Manotas et al. 2020; Raznahan et al. 2018) but are already yielding important new insights that inform our mechanistic understanding of SCAs. Specifically, these studies (San Roman et al. 2023; Zhang et al. 2020; Liu et al. 2023; Raznahan et al. 2018) and our evidence synthesis (Figs.5 and 6), find a core set of sex chromosome genes that appear to show consistent changes in expression with variations in X- and Y-chromosome dosage. Stepwise increases in the expression of these genes with mounting sex chromosome dosage may therefore contribute to phenotypes that appear to become incrementally more severe with carriage of more supernumerary sex chromosomes, such as neurodevelopmental impacts. However, many SCA phenotypes—like height and gonadal impairment—do not directly track chromosome count in this way, making it important to consider both linear and non-linear X and Y dosage effects, while resolving karyotype-specific as well as shared genomic impacts of SCAs.

Second, the focus of molecular research has been mainly centered on blood- and gonad-related tissues. This overrepresentation stems from practical considerations such as blood accessibility and the availability of testicular samples in patients with hypogonadism, but does not mirror the diversity of SCAs clinically impactful phenotypes such as neuropsychiatric, neurodevelopmental, cognitive, endocrine, metabolic and musculoskeletal. The convergence of research on blood and gonadal tissue has yielded some important mechanistic insights into the clinical impacts of SCAs in these tissue types. For example, blood-focused studies have potential relevance to shed light on the increased immunological risk associated with SCAs (Sim et al. 2025; Raznahan et al. 2018; Sharma et al. 2017) and gonadal-focused studies will help to advance our understanding of infertility-related molecular pathways (Lu et al. 2024; McGlacken-Byrne 2023; Willems et al. 2022; Wang et al. 2022; Galdon et al. 2022; Zhao et al. 2020; Winge et al. 2018, 2018). However, these findings do not necessarily extrapolate to other organs. There is growing recognition of the cell-type specific trans effects of SCAs from comparison across different cell lines (Blanton et al. 2024; Liu et al. 2023), so diversification of the tissues included in genomic studies should ideally be accompanied by greater use of single cell rather than bulk tissue approaches and also the modeling of less accessible tissues like the brain. The recent introduction of stem cell models in SCA research (Astro 2024; Luo et al. 2023; Astro et al. 2023, 2022; Panula et al. 2019; Zhang et al. 2013; Ma et al. 2012; Liu et al. 2023) may provide a path to exploring organ and cell-type specific effects, but this work is still in its infancy and would benefit from more systematic consideration and documentation of potential sources of bias and heterogeneity such as genomic instability and erosion of X-chromosome inactivation during reprogramming and differentiation (Mekhoubad et al. 2012; Ronen and Benvenisty 2012; Yoshihara et al. 2019). Also, given that many SCA phenotypes are developmentally dynamic—such those impacting somatic growth (Hanson et al. 2024), gonadal function (Wang et al. 2022), and brain development (Lenroot et al. 2009)—complete mechanistic accounts will need to understand how some tissue and cell specific effects vary with age.

Third, we document a pronounced concentration of SCA studies on measures of gene expression (Fig. 3). This focus leaves many genomic impacts uncharted (see below) and is subject to publication bias, but has made it possible to nominate specific genes that appear to show highly stereotyped and robust dosage sensitivity to SCAs across multiple contexts. Indeed, our analysis (Figs. 5 and 6) specifies several of these recurrent DEGs, which represent the most reproducibly dosage sensitive PAR (*SLC25A6*, *ASMTL*, *GTPBP6*, *ZBED1*), X-linked (*KDM6A*, *ZFX*, *KDM5C*, *PUDP*) and Y-linked (*ZFY*, *RPS4Y1*) genes across studies. These genes exhibit consistent effects across the diverse reported tissues, and are therefore potential candidates for proximal cis-effects of SCAs that could drive broader genomic impacts. Supporting this notion, we show that these recurrent DEGs encode proteins that coalesce into a large network involved in biological processes capable of imparting trans effects on gene expression. We identify some of these trans-effects in the form of functionally enriched proteins in SCAs, such as histones H2AC6 and H2BC12L, which concentrate in PPI networks involved in meiotic synapsis and telomere maintenance (Fig. 5). Our detection of a PPI cluster enriched for genes involved in histone modification, translational initiation and nucleic acid binding [red] is notable given prior evidence for generic effects of diverse aneuploidy in diverse tissue types on protein metabolism (Sheltzer et al. 2012). In particular, KDM6A, KDM5C, and ZFX all participate in chromatin remodeling through post-translational histone modifications, and their loss of function has known impacts in neurodevelopment (Brookes et al. 2015; Faundes et al. 2021; Shepherdson et al. 2024). Further evidence for SCAs potentially impacting broader aspects of genome regulation comes from a second PPI network [blue] enriched for histone meiotic synapsis and telomere maintenance. Having found these core genes and processes that appear to be recurrently impacted by SCAs provides an helpful starting point for experimental study designs capable of formally testing for the causal impact of these core dysregulations on downstream biological processes. This provides an important foundation to investigate how these the dosage sensitive phenotypes could arise from direct effects (e.g. SCL25A6, the top recurrent PAR gene, is negatively correlated QTc interval, known to be shorter in KS and longer in TS [Skakkebæk et al. 2023]), or through downstream effects, eventually influencing phenotypes involved in affected pathways (e.g. the top recurrent X-linked KDM6A and KDM5C, and autosome DDX43, dysregulated in X-disparate contrasts, are involved in chromatin remodelling, which is known to be associated to neurodevelopmental disorders [Mossink et al. 2021]).

Fourth, although the bulk of the work studying genomic impacts of SCAs has considered alterations in mRNA levels for coding genes, there are multiple non-coding transcripts that may also show imbalances (Cimino et al. 2017; Luo et al. 2023; Umair et al. 2022; Ibarra-Ramírez et al. 2020; Winge et al. 2018; Rajpathak et al. 2014; Sui et al. 2012; Rajpathak and Deobagkar 2017; Sun et al. 2017), but remain largely unstudied, including lncRNAs, microRNAs, circRNAs (Fabbri et al. 2019). Many of these transcripts have roles in genome regulation, making them candidate disseminators of SCA impacts across the genome. Relatedly, there is a pressing need for broader research into the epigenetic impacts of SCAs given the apparent absence of studies examining SCA impacts on genomic features such as histone modifications or short range chromatin interactions, and the relative sparsity of studies on chromatin accessibility (Miao et al. 2023) and long-range chromatin interactions (Zhang et al. 2020). The sparsity of studies on chromatin configuration (Miao et al. 2023; Zhang et al. 2020) is especially notable given the significant enrichment of recurrent DEGs in SCAs for this biological process (Fig.6). In particular those available studies show evidence of higher chromatin accessibility and activity in X chromosomes from individuals with 47,XXY karyotype compared to 46,XY and 46,XX (Miao et al. 2023) and similar 3D chromatin conformation of X chromosomes between the 46,XX and 47,XXY karyotype, and between the active X chromosome in 45,X and 46,XX (Zhang et al. 2020). There is also a strong case for continued expansion of research on SCA impacts in DNA methylation (Astro 2024; Berglund et al. 2024; Lu et al. 2024; Wan et al. 2015; Miao et al. 2023; Gutierrez et al. 2022; Fan et al. 2022; Zhang et al. 2020; Nielsen et al. 2020; Skakkebæk et al. 2018; Viana et al. 2014; Rajpathak and Deobagkar 2014; Trolle et al. 2016; Sharma et al. 2015). To date—notwithstanding methodological heterogeneity across studies of DNA methylation—we can find some consistency between the methylation and transcriptomic studies. Interestingly RADX (CXorf57) the only sex chromosome gene invariably downregulated in the X-disparate contrasts shows concordant promoter hypermethylation in 47,XXY versus 46,XY blood samples (Zhang et al. 2020). Similarly HENMT1 an autosomal gene consistently downregulated in PAR- and X-disparate contrasts is hypermethylated in 47,XXY versus 46,XY and 46,XX blood samples (Wan et al., 2015). In contrast, promoter hypermethylation was not reported in any of the upregulated genes in the PAR- and X- or Y-disparate contrasts from iPSCs and fibroblasts (Wan et al., 2015; Zhang et al., 2020; Astro, 2024). These findings could imply distinct regulatory mechanisms across contrast types pointing to promoter hypermethylation in genes suppressed in PAR- and X-disparate contrasts and as expected minimal methylation changes in upregulated genes specially in Y-chromosome dosage disparities (Astro et al., 2025). Given the plurality and interrelatedness of processes regulating gene expression, a pressing priority for future research will be simultaneously integrating information from multiple epigenetic and transcriptomic assays in SCAs.

Fifth, even though transcriptomics—the predominant source of available genomic data—is commonly used as a proxy for inferring functional pathways, the correlation between gene expression, protein levels and function is not perfect (Piran et al. 2020) and almost nothing is known regarding SCA impacts on the proteome. The highly complex translational and post-translational processes that lie downstream of transcription are not captured by measurement of mRNA alone, but are highly likely to be disrupted themselves by SCAs given that several recurrent DEG products are related to translation of mRNA into protein (e.g. EIF2S3, EIF1AY, EIF1AX, DDX3X, RPS4Y1, RPS4X) and post-translational modifications such as demethylation (KDM5D, PHF2, KDM5C, KDM6A, UTY) or deubiquitination (USP9X, UBA1, USP9Y, OTUD6B).

By comprehensively mapping existing knowledge in genomic research in SCAs and outlining salient gaps, our Systematic Review helps to inform the design of future research in several key directions. First, we have identified that future research on SCAs would benefit from more comprehensive inclusion of multiple karyotypes, and a greater range of primary sample types, with incorporation of single cell genomic technologies to resolve cell-type specific effects within organ systems. Second, there is a pressing need to diversify and integrate a wider range of genomic readouts in SCA research to better model how dosage alterations of the X- and Y-chromosome alter genomic regulation to ultimately manifest as proteomic changes underlying disrupted cellular physiology. Third, while expanded genomic datasets will be important to build richer mechanistic models, testing these models will ultimately require experimental perturbations. The field is well equipped for this causal work due to the current availability of: (i) convergent lines of evidence nominating a core set of sex chromosome genes that appear to show almost obligate dosage sensitivity and biological functions capable of driving wider genomic effects; (ii) access to ever-more sophisticated 2D and 3D in vitro systems for modeling cell-autonomous and interactive processes (Sato et al. 2009; Matsa et al. 2016; Kundu et al. 2022); and, (iii) a rapidly expanding toolkit for targeted genome editing. While successfully addressing all of the gaps above would deliver causal models for SCA impacts at the group level, advancing translational medicine of SCAs through genomic insights will ultimately require studies of interindividual variability. Interindividual variation in the expression of dosage-sensitive genes may explain phenotypic heterogeneity and help to stratify patients based on individual profiles (e.g. testing if aneuploidic individuals with larger changes in chromatin related genes could be at greater risk for neurodevelopmental disorders). Also, the intertissue consistency of cis-effects on sex chromosome compartments could theoretically enable blood-based molecular profiling to predict outcomes in harder to access tissues like the brain. Finally, some pathways showing consistent dysregulation could be modulated pharmacologically. Critically linking the molecular findings to clinical outcomes in prospective cohorts will be essential to validate their utility. Reaching for this ambitious goal hinges on our ability to scale up current genomic research in SCAs by orders of magnitude—an effort that will demand dedicated funding of large-scale team science efforts in close partnership and collaboration with people who have lived experience of SCAs.

Taken together, our systematic review gives a comprehensive overview that consolidates existing knowledge from genomic studies in SCAs and provides a strong empirical base for recognizing current challenges and charting pathways to a richer and more useful understanding of sex chromosome influences on the human genome.

## Methods

The protocol for this systematic review was registered on PROSPERO, registration number *CRD42024473984.* The full protocol under PRISMA-P guidelines is available in Supplementary File 4. The PRISMA checklist for this systematic review is in Supplementary File 5.

### Eligibility criteria

Broadly, eligible studies were those on human individuals with sex chromosome aneuploidies (SCAs) and euploid or SCA controls, that used non-interventional approaches to unbiasedly profile genome-wide elements such as gene expression and its regulators. Therefore, we excluded studies on non-human subjects, or those studying a specific set of genes, chromosomes or genomic regions. Studies that included both non-mosaic and mosaic individuals were included, but only non-mosaic participants were counted as eligible and considered for further statistics and analysis. Studies reanalyzing results from previously reported datasets were excluded, and studies considering the same cohort of subjects were merged. The inclusion and exclusion criteria were structured utilizing the Population, Exposure, Comparator, and Outcomes (PECO) framework and are reported in Supplementary Table 1.

### Information sources

The following databases were consulted: Embase, Pubmed, Web of Science, Scopus, ClinicalTrials.gov, Clarivate ProQuest, Clarivate Preprint Citation Index, bioRxiv and medRxiv, for original peer reviewed articles, articles ahead of print, dissertations, thesis, and reports. We also performed reference and citation searches of the retrieved articles.

### Search strategy

The search strategy was reviewed and refined in collaboration with a trained librarian. The search was performed without filtering date, species or language, to avoid missing articles because of mislabeling. The primary search terms were based on the core concepts framework in PECO: ‘Sex Chromosome Aneuploidies’ (Population), ‘Techniques, assays or methods for Gene expression or Transcriptional regulation measurement’ (Exposure), and “Genome Structure and Function” (Outcome). For refining the search terms, we first survey the controlled vocabulary database (i.e MeSH, EMtree) for the term closer to the core concept. We then analyzed the scope and precision of our search results, and expanded or trimmed the search accordingly by adding semantically related terms from the keywords, abstracts, vocabulary hierarchies or standard uses in scientific literature, while excluding redundant or ambiguous terms. The Pubmed refining process was supported by the visual inspection of the overlapping or nesting inter-relation across search terms, with the tool PubVenn (PubVenn 2025). Available from https://pubvenn.appspot.com/*).* This strategy guided the selection of the most comprehensive and specific search terms. A schematic graphical abstract of this approach is in Fig. S1. The full search strategy is available in File S1.

### Selection process

Literature search results from multiple databases were captured into an EndNote 20 Library (Clarivate Analytics) and then exported to Covidence (Veritas Health Innovation, Melbourne, Australia. Available at www.covidence.org, 2025) for inclusion and exclusion review. The built-in Covidence machine learning tool for prioritizing records during screening selection was used. Titles and abstracts were independently screened by two reviewers for eligibility based on the inclusion and exclusion criteria, with a Cohen’s Kappa of 0.77. Disagreements between the two reviewers (MS, ML) were discussed and resolved through consensus. The full texts from candidate articles were assessed for eligibility by the same two reviewers, with a Cohen’s Kappa of 0.74. Reasons for exclusion were recorded. Conflicts were discussed and a third reviewer (AR) was consulted for three articles in which unanimous decision was not reached. After defining eligible articles, forward and backward search of their references and citations was performed. The resulting references were uploaded to Covidence for abstract screening and their eligibility assessed with the same procedure as before. The summary of the selection process according to the PRISMA recommendations is depicted in Fig. 1.

### Data collection

#### Data collection process

Article information of all eligible studies was extracted using a template based on the PECO structure customized to integrate all relevant information necessary for our review. The process was performed by two reviewers (MS, ML) on the Covidence platform. The final data was compared and inconsistencies settled by consensus.

#### Data items

The data items used for summary statistics are listed in Supplementary File 3.

### Bias assessment

#### Risk of bias

We evaluated the risk of bias by applying the ROBINS-E tool, which formalizes potential biases under 6 domains: confounding factors, participant selection, missing data, post-exposure interventions, measurement of outcome effect and bias in selection of the reported results. We operationalized those categories by considering objective variables—such as sample size, study design, adjustment, inclusion of covariates—to adjust for confounding, karyotype confirmation, validation of results, informed consent, competing interests. Detailed information on variables used for categorization and the individual article analysis are detailed in Supplementary File 2.

#### Reporting bias

Studies utilized for synthesis were subject to an additional bias evaluation by applying the “Tool for assessing risk of bias due to missing evidence in a synthesis” (ROB-ME [Page et al. 2023]), (Supplementary File 2).

### Analysis of literature characteristics

The consensus data was downloaded from covidence in CSV format, and imported in R. All included studies were considered for aggregation of descriptive items such as design, participant karyotype, tissue or cell type evaluated, technique or assay selected.

### Aggregated synthesis for Differentially Expressed Genes (DEGs)

To combine information across studies regarding the genomic impacts of SCAs, we screened included studies for lists of impacted genes derived by contrasting groups of disparate sex chromosome dosage which included at least 3 biological replicates. This criterion identified 55 such contrasts − 49 of which were derived from transcriptomics and 6 from methylation measures. We therefore focused on integrating information regarding differentially expressed genes (DEGs) in SCAs as measured by RNAseq, which yielded 49 DEG lists.

For each of the eligible DEG lists, the full list of DEGs were retrieved. The significance threshold for DEG detection was based on the author report, or a FDR of 0.05 if not explicitly stated. Data was imported and analyzed in R and consolidated in a Summarized Experiment object available at the repository. Since different studies used inconsistent nomenclatures, the reported gene names were mapped against the corresponding Ensembl ID Gencode v47 annotation from GRCh38 assembly, using the AnnotationDbi package (Hervé 2017).

The dataset was subseted on comparisons that involved different number of PAR regions or X- or Y-chromosomes, and the DEG frequency (number of times a gene was reported as DEG) was calculated on each subset. DEGs were ranked by proportion of contrasts in which they were reported, independently for contrasts with X, Y or PAR disparity. The DEGs reported in more than 25% of contrasts were used for synthesis and protein-protein interaction (PPI) network construction. The 25% cut-off was determined by evaluating the balance between network density and gene set sizes for downstream analysis. The PPI network was built using interactions reported in the StringDB package in R, with a confidence score of 400 (moderate). The clustering was calculated with the Walktrap algorithm from the Igraph package (Csárdi 2025), and the enrichment analysis was completed using the built-in databases from StringDB R package.

## Supplementary Information

Supplementary Material 1. Bias Assessment.

Supplementary Material 2. Data collection items.

Supplementary Material 3. Table S1. Enrichment list.

Supplementary Material 4. PRISMA protocol.

Supplementary Material 5. PRISMA checklist.

Supplementary Material 6. Search strategy.

Supplementary Material 7. Table S2. Inclusion and exclusion criteria.

Supplementary Material 8. Table S3. DEG list.

Supplementary Material 9. Table S4. Studies characteristics extended and merged studies

Supplementary Material 10. Figures S1-S7.

## Data Availability

Provided in supplementary material.
