# Supplementary material for: Sex chromosome aneuploidy impacts on human gene expression and regulation: a systematic review
Source: Mol Med. 2025 Dec 30;32:11. doi: 10.1186/s10020-025-01404-1 (PMC12859965; doi:10.1186/s10020-025-01404-1)
Supplement: Supplementary file 2 — Supplementary Material 2. Data collection items. [file 10020_2025_1404_MOESM2_ESM.pdf]

Supplementary File 3.
Data collection items.

1. Covidence
2. Study ID
3. Title
4. Title 2
5. Reviewer Name
6. Publication year
7. DOI
8. Country in which the study conducted
9. Other countries if more than one separate with
10. Name
11. Institution
12. Email
13. Aim of study
14. Study design
15. Study funding sources
16. Possible conflicts of interest for study authors
17. Additional study data
18. Inclusion criteria
19. Exclusion criteria
20. Group differences comments
21. Total number of participants
22. Total number of eligible participants
23. Baseline characteristics
24. Mean age years SD Population
25. Mean age years SD Control group
26. Karyotypes separated by Population
27. Karyotypes separated by Control group
28. Sample size Population
29. Sample size Control group
30. Country of origin Population
31. Country of origin Control group
32. Participants karyotypes sex chromosomes
33. XY Number of participants
34. XX Number of participants
35. XO Number of participants
36. XXY Number of participants
37. XXX Number of participants
38. XYY Number of participants
39. XXYY Number of participants
40. XXXY Number of participants

41. XXXX Number of participants
42. XXXXY Number of participants
43. Other high grade aneuploidies Number of participants
44. Are karyotypes citogenetically confirmed
45. Did patients signed informed consent
46. Did this study has Ethics Committee approval
47. Additional population comments
48. Method and Tissue
49. RNA seq Whole blood
50. RNA seq Blood derived
51. RNA seq Fibroblasts
52. RNA seq Gonad derived
53. RNA seq Stem cells
54. RNA seq Other cell lines
55. RNA seq Other describe
56. Expression microarray Whole blood
57. Expression microarray Blood derived
58. Expression microarray Fibroblasts
59. Expression microarray Gonad derived
60. Expression microarray Stem cells
61. Expression microarray Other cell lines
62. Expression microarray Other describe
63. Small RNA targeted RNA seq Whole blood
64. Small RNA targeted RNA seq Blood derived
65. Small RNA targeted RNA seq Fibroblasts
66. Small RNA targeted RNA seq Gonad derived
67. Small RNA targeted RNA seq Stem cells
68. Small RNA targeted RNA seq Other cell lines
69. Small RNA targeted RNA seq Other describe
70. ATAC seq Whole blood
71. ATAC seq Blood derived
72. ATAC seq Fibroblasts
73. ATAC seq Gonad derived
74. ATAC seq Stem cells
75. ATAC seq Other cell lines
76. ATAC seq Other describe
77. Bisulfite sequencing Whole blood
78. Bisulfite sequencing Blood derived
79. Bisulfite sequencing Fibroblasts
80. Bisulfite sequencing Gonad derived
81. Bisulfite sequencing Stem cells
82. Bisulfite sequencing Other cell lines
83. Bisulfite sequencing Other describe
84. DNA methylation array Whole blood

85. DNA methylation array Blood derived
86. DNA methylation array Fibroblasts
87. DNA methylation array Gonad derived
88. DNA methylation array Stem cells
89. DNA methylation array Other cell lines
90. DNA methylation array Other describe
91. Single cell RNA seq Whole blood
92. Single cell RNA seq Blood derived
93. Single cell RNA seq Fibroblasts
94. Single cell RNA seq Gonad derived
95. Single cell RNA seq Stem cells
96. Single cell RNA seq Other cell lines
97. Single cell RNA seq Other describe
98. Single cell ATAC seq Whole blood
99. Single cell ATAC seq Blood derived
100. Single cell ATAC seq Fibroblasts
101. Single cell ATAC seq Gonad derived
102. Single cell ATAC seq Stem cells
103. Single cell ATAC seq Other cell lines
104. Single cell ATAC seq Other describe
105. Other describe Whole blood
106. Other describe Blood derived
107. Other describe Fibroblasts
108. Other describe Gonad derived
109. Other describe Stem cells
110. Other describe Other cell lines
111. Other describe Other describe
112. Tissue additional comments
113. Is the analysis sex aware
114. Is the analysis allele specific
115. Other intervention related comments
116. Genomic component targeted
117. If other describe
118. Does the publication include DEGs or DMR list
119. Does the publication include GO enrichment list
120. Were results validated by laboratory
121. Additional outcome related comments
122. Intervention and sample processing comments
123. RNA seq Library kit
124. RNA seq Sequencing platform
125. RNA seq Additional details
126. Expression microarray Library kit
127. Expression microarray Sequencing platform
128. Expression microarray Additional details

129. Small RNA targeted RNA seq Library kit
130. Small RNA targeted RNA seq Sequencing platform
131. Small RNA targeted RNA seq Additional details
132. ATAC seq Library kit
133. ATAC seq Sequencing platform
134. ATAC seq Additional details
135. Bisulfite sequencing Library kit
136. Bisulfite sequencing Sequencing platform
137. Bisulfite sequencing Additional details
138. DNA methylation array Library kit
139. DNA methylation array Sequencing platform
140. DNA methylation array Additional details
141. Single cell RNA seq Library kit
142. Single cell RNA seq Sequencing platform
143. Single cell RNA seq Additional details
144. Single cell ATAC seq Library kit
145. Single cell ATAC seq Sequencing platform
146. Single cell ATAC seq Additional details
147. Other Library kit
148. Other Sequencing platform
149. Other Additional details
150. Intervention and bioinformatic analysis R1
151. RNA seq QC tool
152. RNA seq Trimming tool
153. RNA seq Alignment tool
154. RNA seq Differential expression analysis tool
155. RNA seq Additional comments
156. Expression microarray QC tool
157. Expression microarray Trimming tool
158. Expression microarray Alignment tool
159. Expression microarray Differential expression analysis tool
160. Expression microarray Additional comments
161. Small RNA targeted RNA seq QC tool
162. Small RNA targeted RNA seq Trimming tool
163. Small RNA targeted RNA seq Alignment tool
164. Small RNA targeted RNA seq Differential expression analysis tool
165. Small RNA targeted RNA seq Additional comments
166. ATAC seq QC tool
167. ATAC seq Trimming tool
168. ATAC seq Alignment tool
169. ATAC seq Differential expression analysis tool
170. ATAC seq Additional comments
171. Bisulfite sequencing QC tool
172. Bisulfite sequencing Trimming tool

173. Bisulfite sequencing Alignment tool
174. Bisulfite sequencing Differential expression analysis tool
175. Bisulfite sequencing Additional comments
176. DNA methylation array QC tool
177. DNA methylation array Trimming tool
178. DNA methylation array Alignment tool
179. DNA methylation array Differential expression analysis tool
180. DNA methylation array Additional comments
181. Single cell RNAseq QC tool
182. Single cell RNAseq Trimming tool
183. Single cell RNAseq Alignment tool
184. Single cell RNAseq Differential expression analysis tool
185. Single cell RNAseq Additional comments
186. Single cell ATAC seq QC tool
187. Single cell ATAC seq Trimming tool
188. Single cell ATAC seq Alignment tool
189. Single cell ATAC seq Differential expression analysis tool
190. Single cell ATAC seq Additional comments
191. Other QC tool
192. Other Trimming tool
193. Other Alignment tool
194. Other Differential expression analysis tool
195. Other Additional comments
196. Gene expression changes
197. Number of DEGS Value
198. Number of DMR Value
199. Functional changes
200. Molecular function Number of enriched terms
201. Molecular function Top 1 enriched
202. Molecular function Top 2 enriched
203. Molecular function Top 3 enriched
204. Molecular function Comments
205. Biological process Number of enriched terms
206. Biological process Top 1 enriched
207. Biological process Top 2 enriched
208. Biological process Top 3 enriched
209. Biological process Comments
210. Cellular component Number of enriched terms
211. Cellular component Top 1 enriched
212. Cellular component Top 2 enriched
213. Cellular component Top 3 enriched
214. Cellular component Comments
215. Reactome pathway Number of enriched terms
216. Reactome pathway Top 1 enriched

217. Reactome pathway Top 2 enriched
218. Reactome pathway Top 3 enriched
219. Reactome pathway Comments
220. KEGG pathway Number of enriched terms
221. KEGG pathway Top 1 enriched
222. KEGG pathway Top 2 enriched
223. KEGG pathway Top 3 enriched
224. KEGG pathway Comments
225. Wikipathway Number of enriched terms
226. Wikipathway Top 1 enriched
227. Wikipathway Top 2 enriched
228. Wikipathway Top 3 enriched
229. Wikipathway Comments
230. TRANSFAC regulatory motifs Number of enriched terms
231. TRANSFAC regulatory motifs Top 1 enriched
232. TRANSFAC regulatory motifs Top 2 enriched
233. TRANSFAC regulatory motifs Top 3 enriched
234. TRANSFAC regulatory motifs Comments
235. mirTarBase regulatory motifs Number of enriched terms
236. mirTarBase regulatory motifs Top 1 enriched
237. mirTarBase regulatory motifs Top 2 enriched
238. mirTarBase regulatory motifs Top 3 enriched
239. mirTarBase regulatory motifs Comments
240. Human Phenotype Ontology database Number of enriched terms
241. Human Phenotype Ontology database Top 1 enriched
242. Human Phenotype Ontology database Top 2 enriched
243. Human Phenotype Ontology database Top 3 enriched
244. Human Phenotype Ontology database Comments
245. Other Number of enriched terms
246. Other Top 1 enriched
247. Other Top 2 enriched
248. Other Top 3 enriched
249. Other Comments
