# Supplementary material for: Sex chromosome aneuploidy impacts on human gene expression and regulation: a systematic review
Source: Mol Med. 2025 Dec 30;32:11. doi: 10.1186/s10020-025-01404-1 (PMC12859965; doi:10.1186/s10020-025-01404-1)
Supplement: Supplementary file 4 — Supplementary Material 4. PRISMA protocol. [file 10020_2025_1404_MOESM4_ESM.pdf]

Systematic Review Protocol (Based on PRISMA-P)

ADMINISTRATIVE INFORMATION

1. **Title**

a. **Identification:** Genome structure and function in individuals with sex chromosome aneuploidies: Protocol for a systematic review.

b. **Update:** First time protocol, not an update.

2. Registration

In accordance with the PRISMA guidelines, our systematic review protocol will be registered with the International Prospective Register of Systematic Reviews (PROSPERO)

3. Authors

a. Contact

Corresponding author: Dr Raznahan, Armin. E-mail *raznahana@nih.gov* . Section on Developmental Neurogenomics, Human Genetics Branch, National Institute of Mental Health, National Institute of Health, 10 Center Drive, Room 4N242, Bethesda, MD.

Mastronardo, Maya. E-mail: *mmastro5@jh.edu*. Section on Developmental Neurogenomics, Human Genetics Branch, National Institute of Mental Health, National Institute of Health, 10 Center Drive, Room 4N242, Bethesda, MD. Johns Hopkins Cell Biology, Molecular Biology, Developmental Biology and Biophysics department in the Krieger School of Arts and Sciences.

Legue, Marcela. E-mail: *marcela.legue@nih.gov* . Section on Developmental Neurogenomics, Human Genetics Branch, National Institute of Mental Health, National Institute of Health, Bethesda, MD.

Cooper, Diane. Email: diane.cooper@nih.gov . Office of Research Services, NIH Library, National Institutes of Health, Bethesda, MD.

Butera, Gisela. Email: gisela.butera@nih.gov . Office of Research Services, NIH Library, National Institutes of Health, Bethesda, MD.

Melanie Staszewski. Email: melanie.staszewski@nih.gov. Section on Developmental Neurogenomics, Human Genetics Branch, National Institute of Mental Health, National Institute of Health, Bethesda, MD.

b. Contribution

AR is the guarantor. AR, MM, ML, GB designed the protocol, and defined eligibility criteria and data extraction criteria. GB, MM, and ML developed the search strategy. AR, MS and ML performed screening, article selection, data collection and statistical analysis. All the authors contributed to writing the manuscript, provided feedback and approved the final manuscript.

4. Amendments

Not applicable.

5. Support - sources

The authors completed this work as part of the Intramural Research Program of the National Institute of Mental Health.

INTRODUCTION

Background

6. Rationale

Overview.

Sex chromosome aneuploidies (SCA) are conditions defined by an atypical number of sex chromosomes, different from XX or XY. The most frequent SCA are XO, XXY, XXX, XYY and XXYY. The clinical impact of SCA varies according to karyotype, but all of them increase the likelihood of neurodevelopmental difficulties and have also consequences on other organ systems. These manifestations must ultimately stem from proximal effects of altered Sex Chromosome Dosage (SCD) on gene regulation and cellular organization.

While brain mechanisms for SCA associations with neurodevelopmental and psychiatric associations are still unclear, there is robust evidence SCA alters human brain structure and function (Hong et al., 2014; Lepage et al., 2014; Mankiw et al., 2017; Nadig et al., 2018;

Raznahan et al., 2016; Whitman et al., 2021), with some effects being divergent between the X- and Y-chromosomes (e.g. effects on total brain volume) and some convergent (e.g. effects on regional brain volume) (Raznahan, 2016). It is also clear that particular brain regions are more vulnerable to SCA effects on their structure and function than others (Hong et al., 2014; Lepage et al., 2014; Mankiw et al., 2017).

We do not currently understand the molecular mechanisms that drive the emergence of the complex phenotypic outcomes in the brain (or any other organ) from changes in SCD. Understanding these effects is not only important for mechanistic understanding of SCAs as neurogenetic disorders, but may also provide a window to better understand X chromosome contribution to neurodevelopment (Mallard 2021) and to unveil SCD contributions to normative sex differences between XY males and XX females (Arnold & Chen, 2009)* Despite these strong motivations from both clinical and basic science for understanding the proximal effects of SCD on the human genome function, the corpus of studies on this topic remains limited. There have been rapid recent advances in tools for cellular reprogramming of human tissues (Chen et al., 2011) and measuring human genome structure and function at high throughput (Gasperskaja & Kučinskas, 2017). This holds a great promise in application to the study of SCD effects (Raznahan et al., 2018; Zhang et al., 2020)). It is therefore an opportune time to take stock of what is already known and what questions should be prioritized for future work on the genomic and cellular effects of SCD variation.

Overall, there is a general association between increasing sex chromosome dosage (SCD) and mounting risk for cognitive impairments and psychopathology (Bishop et al., 2011; Cordeiro et al., 2012; Green et al., 2019; Hong & Reiss, 2014; Rau et al., 2021; Sánchez et al., 2023; N. R. Tartaglia et al., 2010; Wilson et al., 2019). There is also some evidence, however, that the profile of neurodevelopmental features may vary between SCA subtypes. For example, population-based study designs that minimize ascertainment bias (Sánchez et al., 2023) suggest XYY may increase risk for ASD and ADHD more than XXY, and that XO increases risk for ASD more than both male trisomies. Conversely, trisomy X can substantially increase the likelihood of bipolar affective disorders to an extent not seen in other trisomic SCAs (Sánchez et al., 2023).

Relevance.

7. Objectives

Our aim is to understand the effects on genome structure and function of sex chromosome dosage variations and its impact in cellular process in individuals with sex chromosome aneuploidies. The PECO components of the review objective are as follows:

Patient/Population: Individuals (human) with sex chromosome aneuploidies.

Exposure: Techniques, assays or methods designed for unbiased measurement of gene expression or transcriptional regulation.

Comparison: Controls without aneuploidy or with a different type of sex chromosome aneuploidy.

Outcome: Impact on genome structure and function evidenced through changes in pattern of gene expression or regulatory mechanisms at the transcriptional level.

METHODS

8. Eligibility Criteria

Population

Inclusion:

- Individuals with sex chromosome aneuploidies.

Exclusion:

- Individuals with mosaic sex chromosome aneuploidies.
- Individuals with partial sex chromosome deletions (e.g., 46,XYq- karyotype) or with sex chromosome microdeletions.
- Sex chromosome aneuploidy on cells or tissues of somatic origin, such as in cancer.
- Aneuploid cells or tissues that involve autosomal chromosomes, such as polyploid or non-diploid cells.
- Use of animal models only.
- X chromosome-related disorders (i.e Fragile X syndrome)

Exposure

Inclusion:

- Techniques, assays or methods designed for unbiased or genome-wide measurement of gene expression or transcriptional regulation mediators.

- Studies performed can include genomic, transcriptomic, epigenomic, or any other technique designed for evaluating genome structure or function.
- Samples are tissues or cells from individuals with sex chromosome aneuploidies.

Exclusion:

- Techniques, assays or methods that only evaluate single nucleotide or copy number variations (such as whole genome sequencing, exome sequencing or comparative genomic hybridization)
- Studies focused on a single gene or small pre-defined set of genes or loci. Some examples of techniques designed for specific genes are Polymerase chain reaction (PCR), Real time PCR (RT-PCR) or quantitative RT-PCR (qRT-PCR), Northern Blot, Southern blot, Western Blot, Gene-specific methylation.

Comparison

Inclusion:

- Healthy controls or individuals with a different sex chromosome aneuploidy.

Exclusion:

- Individuals with mosaic sex chromosome aneuploidies.
- Individuals with partial sex chromosome deletions (ej: 46,XYq- karyotype) or with sex chromosome microdeletions.

Outcome

Inclusion:

- Measures genome structure and function evidenced through changes in pattern of gene expression or regulatory mechanisms at the transcriptional level.

Exclusion:

- Evaluation circumscribed to a specific gene or a small set of genes.
- Measures not related to gene expression or regulatory mechanisms. Examples of unrelated measures are specific treatments, prenatal screening, or unrelated diagnostic tests.

9. Information sources

Scientific databases to be searched for original peer reviewed articles include:

A. Electronic databases of scientific literature

- PubMed -MEDLINE
- EMBASE
- Web of Science
- Scopus

B. Other sources we will look for clinical trials, articles ahead of print, dissertations, thesis, or reports:

- Clinicaltrials.gov
- Preprint citation index
- Biorxiv
- Medrxiv
- NDLTD global ETD search

10. Search strategy

The search terms were selected following the core concepts of "Sex Chromosome Aneuploidies"(population) , techniques, assays or methods designed for unbiased measurement of "Gene Expression or Transcriptional Regulation" (exposure), and impact on "Genome Structure and Function" evidenced through changes in pattern of gene expression or regulatory mechanisms at the transcriptional level.

Based on the core concepts described, we defined search terms and built the search strategy by following the next steps. First, we looked for the closest term related to the core concept at the controlled vocabulary database (i.e MeSH, EMtree). Then, we analyzed their search results and expanded the search including semantically related terms, found on keywords, abstracts, and from standard use in scientific literature. For Pubmed results we performed a visual inspection of the connection and overlapping of article retrieval with each term utilizing the tool PubVenn (https://pubvenn.appspot.com/). This inspection allowed us to define which added terms were useful for exhaustiveness, and which redundant terms to exclude. An example of this approach is in S.Fig. X. The search strategy was reviewed and refined by a librarian.

.

(The search filter excludes articles non-human focused and articles in other languages than English.We will include peer-reviewed original research articles, preprint research articles and original research thesis or dissertations in language (will allow direct untranslated application of

text mining tools). We will exclude reviews, books or retracted articles. No time restriction will be applied.)

The detailed example of the search strategy for Pubmed / MEDLINE is in Appendix 1

11. Study records

a. Data management.

Literature search results from multiple databases will be captured into an EndNote 20 Library (Clarivate Analytics) and then exported to Covidence for inclusion and exclusion review.

b. Selection process

We will use the platform Covidence for managing the selection process. We will use the Covidence machine learning built-in tool for prioritising records during screening selection. Titles and abstracts will be first independently screened by two reviewers for eligibility based on the inclusion and exclusion criteria, reasons for exclusion will be recorded. Any disagreements between the two reviewers (MM, ML) will be discussed and resolved through consensus. A third reviewer (AR) will be consulted if a unanimous decision is not reached. Full text from candidate eligible articles will be assessed for eligibility confirmation by the same two reviewers. After defining eligible articles, we will perform both forward and backward search of their references and citations. The references will be retrieved and prioritised and their eligibility assessed with the same procedure as before.

We will create a flowchart following the PRISMA recommendations for depicting this process.

c. Data collection process

The data extraction will be performed with aid of Covidence software from full-text eligible articles. The process will be performed by two reviewers, and collected in a tabular form, for each item below. The data will be compared and inconsistencies settled by a third reviewer.

12. Data items

The following data will be extracted from eligible articles that met the inclusion criteria:

- Article metadata (Authors, year, journal, ID, DOI)
- Study design
- Participant(s) karyotype(s)
- Sample size (for each karyotype)
- Tissue or cell type analysed
- Type of genomic assay performed (RNAseq, Microarray, ATACseq, BSseq, Chipseq, others)
- Sample processing technical details (kits, sequencing platforms)
- Bioinformatic analysis details (pipeline if applicable, software for each step, use of sex-chromosome informed genomic references or downstream analysis)
- Type of genomic component targeted (Coding RNAs, Non-coding RNAs, DNA Methylation, Histone modification, Chromatin structure, others)
- Genomic functional analysis (if aplicable sets of genes differentially expressed and their metrics (Log2FC and p), differentially methylated regions, chromatin accessibility, results validation, other reported outcomes)

  Any missing or incomplete data from included studies of the respective author(s) will be contacted for further detailed information.

13. Outcomes and prioritization

The primary outcome will be the recognition and characterization of changes in genome structure and function in individuals with sex chromosome aneuploidies. This outcome will be measured as changes in pattern of gene expression or regulatory mechanisms at the transcriptional level, such as methylated regions, histone modifications, chromatin structure, and the functional enriched pathways reported if available.

The secondary outcome will be to pinpoint the methodological aspects and the studies analysed.

14. Risk of bias in individual studies

The quality of individual studies will be assessed following the Cochrane tool "Risk Of Bias In Non-randomised Studies- of Interventions (ROBINS-I). We will perform a checklist for each study, scoring items by two independent reviewers. Individual study scores will be calculated to define the quality high or low risk of bias. Disagreements will be resolved by discussion between the reviewers.

15. Data synthesis

a. Describe criteria under which study data will be quantitatively synthesised

For data synthesis , we will select articles with at least three biological or technical replicates.

If there are more than 5 studies for a given combination of aneuploidy type, method of analysis, and tissue or cell type, we will perform quantitative synthesis in that subgroup

b. If data are appropriate for quantitative synthesis, describe planned summary measures, methods of handling data and methods of combining data from studies, including any planned exploration of consistency (such as I2, Kendall's τ)

We will perform a quantitative synthesis and qualitative review of the characteristics of the articles and their results.

First, we will generate summary tables and plots of descriptive statistics regarding publication trends (number of articles across the years), number and percents of karyotypes included, number and percent of source of tissues or cell types studied, type and genomic component targeted by the genomic assay performed, name of bioinformatic tools applied and if references were informed by the presence or absence of Y chromosomes on samples.

Second, for each type of genomic component targeted (Coding RNAs, Non-coding RNAs, DNA Methylation, Histone modification, Chromatin structure, others), we will aggregate data systematically if the sample size, evaluation technique and source of samples are comparable. To do so, we will use the method of combining p- values and summarising effect estimates, as recommended in the Cochrane Handbook for Systematic Reviews of Interventions. For gene expression data, we will generate an aggregate report of genes differentially expressed, with the magnitude and direction of change across karyotypes. For each other type of genomic component, we will report the features implicates and their magnitude and direction when applicable (such as differentially methylated regions, chromatin accessibility, etc). If previous data is homogeneous enough for statistical testing, we will perform enrichment and pathways analysis of reported genes. In the case that the heterogeneity of methods, sources or small sample sizes preclude us from a quantitative systematic synthesis, we will report a qualitative review of the main results and descriptive statistics.

16. Meta-bias

We are aware of publication bias and outcome reporting bias and we will consider it in the discussion. No specific tool for gene expression or genome structure is applicable in this context.

17. Confidence in cumulative evidence

We will summarise the confidence in our results using the Grading of Recommendations Assessment, Development and Evaluation (GRADE) approach.
