# Supplementary material for: Sex chromosome aneuploidy impacts on human gene expression and regulation: a systematic review
Source: Mol Med. 2025 Dec 30;32:11. doi: 10.1186/s10020-025-01404-1 (PMC12859965; doi:10.1186/s10020-025-01404-1)
Supplement: Supplementary file 6 — Supplementary Material 6. Search strategy. [file 10020_2025_1404_MOESM6_ESM.pdf]

NIH Library Search Request

Requested By: Legue, Marcela (NIMH) | marcela.legue@nih.gov; Raznahan, Armin (NIMH) | raznahana@mail.nih.gov; Mastronardo, Maya (NIMH) | maya.mastronardo@nih.gov

Databases to search:

- PubMed/MEDLINE (National Library of Medicine)
- Embase (Elsevier)
- Web of Science: Core Collection (Clarivate Analytics)
- Scopus (Elsevier)

Grey Literature:

- ClinicalTrials.gov
- Preprint citation index (Clarivate)
- Biorxiv
- Medrxiv
- ProQuest Theses and Dissertations (NDLTD)

Database: PubMed/MEDLINE
Platform: National Library of Medicine
Date Searched: 1/18/2024
Updated Search: 2/26/2025

	Concept:	Search Strategy:
#1	Sex Chromosomes	"Sex Chromosome Aberrations"[MeSH Terms] OR (("Sex Chromosomes"[MeSH Terms] OR "sex chromosom*"[Title/Abstract] OR "x chromosom*"[Title/Abstract] OR "y chromosom*"[Title/Abstract]) AND ("Aneuploidy"[MeSH Terms] OR "aneuploid*"[Title/Abstract] OR "aberration*"[Title/Abstract] OR "abnormalit*"[Title/Abstract] OR "variation*"[Title/Abstract] OR "dosage"[Title/Abstract] OR "alteration*"[Title/Abstract])) OR "Turner Syndrome"[MeSH Terms] OR "turner syndrome*"[Title/Abstract] OR "X monosomy"[Title/Abstract:~2] OR "X0 karyotype"[Title/Abstract:~2] OR "Klinefelter Syndrome"[MeSH Terms] OR "klinefelter syndrome*"[Title/Abstract] OR "XXY"[Title/Abstract] OR "XXYY"[Title/Abstract] OR "XYY"[Title/Abstract] OR "Triple X syndrome"[Title/Abstract:~2] OR "Triple X syndromes"[Title/Abstract:~2] OR "47 XXX"[Title/Abstract:~2] OR "extra X chromosome"[Title/Abstract:~4] OR "extra X chromosomes"[Title/Abstract:~4]

#2	Gene Expression transcriptional regulation (Non-interventional Evaluation)	"Gene Expression Profiling"[Mesh] OR "gene expression profiling"[Title/Abstract] OR "Oligonucleotide Array Sequence Analysis"[Mesh] OR "expression microarray"[Title/Abstract:~3] OR "expression microarrays"[Title/Abstract:~3] OR "High-Throughput Nucleotide Sequencing"[Mesh] OR "high-throughput sequencing"[Title/Abstract] OR "next generation sequenc*"[Title/Abstract] OR "deep sequencing"[Title/Abstract] OR "pyrosequencing"[Title/Abstract] OR "illumina sequencing"[Title/Abstract] OR "ribosome profiling"[Title/Abstract] OR "Sequence Analysis, RNA"[Mesh] OR "RNA Sequencing"[Title/Abstract:~2] OR "RNA-Seq"[Title/Abstract:~2] OR "RNAseq"[Title/Abstract] OR ("transcriptom*"[All Fields] AND "analy*"[All Fields]) OR "ChIP-seq"[Title/Abstract:~2] OR "ChIPseq"[Title/Abstract] OR "chromatin immunoprecipitation sequencing"[Title/Abstract:~3] OR "ATAC-seq"[Title/Abstract:~3] OR "Transposase-Accessible Chromatin"[Title/Abstract:~3] OR "Single-Cell Analysis"[Mesh] OR "single cell analysis"[Title/Abstract:~3] OR "single cell sequencing"[Title/Abstract:~3] OR "scRNA-seq" [Title/Abstract:~2] OR "scATAC-seq" [Title/Abstract:~2] OR "snRNA-seq"[Title/Abstract:~2] OR "bisulfite sequencing"[Title/Abstract:~3] OR "bisulphite sequencing "[Title/Abstract:~3] OR "WGBS"[Title/Abstract] OR "BS-seq"[Title/Abstract:~2] OR "methylated DNA immunoprecipitation"[Title/Abstract:~3] OR "meDIP seq"[Title/Abstract:~2] OR "methyl CpG binding domain sequencing"[Title/Abstract:~4] OR "MBD seq"[Title/Abstract:~3] OR "MRE seq"[Title/Abstract:~2] OR "methylation array"[Title/Abstract:~2] OR "methylation microarray"[Title/Abstract:~2] OR "Hi-C"[Title/Abstract] OR "chromosome conformation capture"[Title/Abstract:~3] OR "chromatin conformation capture"[Title/Abstract:~3] OR "nucleosome mapping"[Title/Abstract:~2] OR "MNase-seq" [Title/Abstract:~3] OR "FAIRE-seq"[Title/Abstract:~3] OR "DNAse-seq"[Title/Abstract:~3] OR "HiChIP"[Title/Abstract] OR "HiCTMap"[All Fields] OR "MeRIP-seq"[Title/Abstract:~2] OR "m6A seq"[Title/Abstract:~2] OR "transcriptom*"[Title/Abstract] OR "Multiomics"[Mesh] OR "multiom*"[Title/Abstract] OR "DNA methylation profil*"[Title/Abstract] OR "DNAm profil*"[Title/Abstract] OR "DNA methylom*"[Title/Abstract] OR "RNA expression profil*"[Title/Abstract]

#3	Gene Expression Regulatory (Mechanisms)	"Gene Expression Regulation"[Mesh] OR "gene expression"[Text Word] OR "gene expression"[Title/Abstract:~2] OR (("gene"[Text Word] AND "expression"[Text Word]) AND ("profile"[Text Word] OR "pattern*"[Text Word])) OR "Gene Regulatory Networks"[Mesh] OR "gene regulation"[Title/Abstract:~2] OR ("differentially expressed"[Title/Abstract:~2] AND "gene"[Text Word]) OR "Transcription, Genetic"[Mesh] OR "genetic transcription"[Title/Abstract:~2] OR "transcriptional regulation"[Title/Abstract:~2] OR "RNA expression"[Title/Abstract:~2] OR "Epigenomics"[Mesh] OR "epigen*"[Title/Abstract] OR "DNA Methylation"[Mesh] OR "differentially methylated regions"[Title/Abstract:~3] OR "differentially methylated region"[Title/Abstract:~3] OR "Chromatin"[Mesh] OR "Histones"[Mesh] OR "nucleosome positioning"[Title/Abstract:~3] OR "DNA binding protein"[Title/Abstract:~2] OR "DNA binding proteins"[Title/Abstract:~2] OR "Transcription Factors"[Mesh] OR "transcriptional factor"[Title/Abstract:~2] OR "transcriptional factors"[Title/Abstract:~2] OR "MicroRNAs"[Mesh] OR "MicroRNAs"[Title/Abstract]
#4	Limits & Filters	(((#1 AND #2 AND #3)

Database: Embase
Platform: Elsevier
Date Searched: 1/18/2024
Updated Search: 2/26/2025

	Concept:	Search Strategy:
#1	Sex Chromosomes	'sex chromosome aberration'/exp OR (('sex chromosome'/exp OR 'sex chromosom*':ti,ab,kw OR 'x chromosom*':ti,ab,kw OR 'y chromosom*':ti,ab,kw) AND ('aneuploidy'/exp OR 'aneuploid*':ti,ab,kw OR 'aberration*':ti,ab,kw OR 'abnormalit*':ti,ab,kw OR 'variation*':ti,ab,kw OR 'dosage':ti,ab,kw OR 'alteration*':ti,ab,kw)) OR 'turner syndrome'/exp OR 'turner syndrome*':ti,ab,kw OR (('x' NEAR/2 'monosomy'):ti,ab,kw) OR (('x0' NEAR/2 'karyotype'):ti,ab,kw) OR 'klinefelter syndrome'/exp OR 'klinefelter syndrome*':ti,ab,kw OR 'xxy':ti,ab,kw OR 'xxyy':ti,ab,kw OR 'xyy':ti,ab,kw OR (('triple' NEAR/2 'x syndrome*'):ti,ab,kw) OR (('47' NEAR/2 'xxx'):ti,ab,kw) OR (('extra' NEAR/4 'x chromosome*'):ti,ab,kw)

#2	Gene Expression transcriptional regulation (Non-interventional Evaluation)	'gene expression profiling'/exp OR 'gene expression profiling':ti,ab,kw OR 'dna microarray'/exp OR (('expression' NEAR/3 'microarray*'):ti,ab,kw) OR 'high throughput sequencing'/exp OR 'high-throughput sequencing':ti,ab,kw OR 'next generation sequenc*':ti,ab,kw OR 'deep sequencing':ti,ab,kw OR 'pyrosequencing':ti,ab,kw OR 'illumina sequencing':ti,ab,kw OR 'ribosome profiling':ti,ab,kw OR 'rna sequencing'/exp OR (('rna' NEAR/2 'sequencing'):ti,ab,kw) OR 'rna-seq' OR 'rnaseq':ti,ab,kw OR ('transcriptom*' AND 'analy*') OR 'chip-seq' OR 'chipseq':ti,ab,kw OR (('chromatin' NEAR/3 'immunoprecipitation sequencing'):ti,ab,kw) OR 'atac-seq' OR (('transposase-accessible' NEAR/3 'chromatin'):ti,ab,kw) OR 'single cell analysis'/exp OR (('single' NEAR/3 'cell analysis'):ti,ab,kw) OR (('single' NEAR/3 'cell sequencing'):ti,ab,kw) OR 'scrna-seq' OR 'scatac-seq' OR 'snrna-seq' OR (('bisulfite' NEAR/3 'sequencing'):ti,ab,kw) OR (('bisulphite' NEAR/3 'sequencing'):ti,ab,kw) OR 'wgbs':ti,ab,kw OR 'bs-seq' OR (('methylated' NEAR/3 'dna mmunoprecipitation'):ti,ab,kw) OR (('medip' NEAR/3 'seq'):ti,ab,kw) OR (('methyl' NEAR/4 'cpg binding domain sequencing'):ti,ab,kw) OR (('mbd' NEAR/3 'seq'):ti,ab,kw) OR (('mre' NEAR/2 'seq'):ti,ab,kw) OR (('methylation' NEAR/2 'array'):ti,ab,kw) OR (('methylation' NEAR/2 'microarray'):ti,ab,kw) OR 'hi-c':ti,ab,kw OR (('chromosome' NEAR/3 'conformation capture'):ti,ab,kw) OR (('chromatin' NEAR/3 'conformation capture'):ti,ab,kw) OR (('nucleosome' NEAR/2 'mapping'):ti,ab,kw) OR 'mnase-seq' OR 'faire-seq' OR 'dnase-seq' OR 'hichip':ti,ab,kw OR 'hictmap' OR 'merip-seq' OR (('m6a' NEAR/3 'seq'):ti,ab,kw) OR 'transcriptom*':ti,ab,kw OR 'multiomics'/exp OR 'multiom*':ti,ab,kw OR 'dna methylation profil*':ti,ab,kw OR 'dnam profil*':ti,ab,kw OR 'dna methylom*':ti,ab,kw OR 'rna expression profil*':ti,ab,kw

#3	Gene Expression Regulatory (Mechanisms)	'gene expression regulation'/exp OR 'gene expression':ti,ab,kw OR (('gene' NEAR/2 'expression'):ti,ab,kw) OR ('gene':ti,ab,kw AND 'expression':ti,ab,kw AND ('profile':ti,ab,kw OR 'pattern*':ti,ab,kw)) OR 'gene regulatory network'/exp OR (('gene' NEAR/2 'regulation'):ti,ab,kw) OR ((('differentially' NEAR/2 'expressed'):ti,ab,kw) AND 'gene':ti,ab,kw) OR 'genetic transcription'/exp OR (('genetic' NEAR/2 'transcription'):ti,ab,kw) OR (('transcriptional' NEAR/2 'regulation'):ti,ab,kw) OR (('rna' NEAR/2 'expression'):ti,ab,kw) OR 'epigenetics'/exp OR 'epigen*':ti,ab,kw OR 'dna methylation'/exp OR (('differentially' NEAR/3 'methylated' NEAR/3 'regions'):ti,ab,kw) OR (('differentially' NEAR/3 'methylated' NEAR/3 'region'):ti,ab,kw) OR 'chromatin'/exp OR 'histone'/exp OR (('nucleosome' NEAR/3 'positioning'):ti,ab,kw) OR (('dna' NEAR/2 'binding' NEAR/2 'protein'):ti,ab,kw) OR (('dna' NEAR/2 'binding' NEAR/2 'proteins'):ti,ab,kw) OR 'transcription factor'/exp OR (('transcriptional' NEAR/2 'factor'):ti,ab,kw) OR (('transcriptional' NEAR/2 'factors'):ti,ab,kw) OR 'microrna'/exp OR 'micrornas':ti,ab,kw
#4	Limits & Filters	(#1 AND #2 AND #3)

Database: Scopus
Platform: Elsevier
Date Searched: 1/18/2024
Updated Search: 2/26/2025

	Concept:	Search Strategy:
#1	Sex Chromosomes	TITLE-ABS-KEY ({Sex Chromosome Aberration} OR (({sex chromosome} OR {x chromosome} OR {y chromosome}) AND ({aneuploid} OR {aberration} OR {abnormalities} OR {variation} OR {dosage} OR {alteration})) OR {turner syndrome} OR {X monosomy} OR {X0 karyotype} OR {klinefelter syndrome} OR {XXY} OR {XXYY} OR {XYY} OR {Triple X syndrome} OR {Triple X syndromes} OR {47 XXX} OR {extra X chromosome})

#2	Gene Expression transcriptional regulation (Non-interventional Evaluation)	TITLE-ABS-KEY ({gene expression profiling} OR {expression microarray} OR {high-throughput sequencing} OR {next generation sequencing} OR {deep sequencing} OR pyrosequencing OR {illumina sequencing} OR {ribosome profiling} OR {rna sequencing} OR {rna seq} OR rnaseq OR (transcriptome AND analysis) OR hictmap OR {chip seq} OR chipseq OR {chromatin immunoprecipitation sequencing} OR {atac seq} OR {transposase accessible chromatin} OR {single cell analysis} OR {single cell sequencing} OR {scrna seq} OR {scatac eq} OR {snrna seq} OR {bisulfite sequencing} OR wgbs OR {bs seq} OR {methylated dna immunoprecipitation} OR {medip seq} OR {methyl cpg binding domain sequencing} OR {mbd seq} OR {mre seq} OR {methylation array} OR {methylation microarray} OR hi-c OR {chromosome conformation capture} OR {chromatin conformation capture} OR {nucleosome mapping} OR {mnase seq} OR {faire seq} OR {dnase seq} OR hichip OR {merip seq} OR {m6a seq} OR transcriptome OR multiome OR {DNA methylation profile} OR {DNAm profile} OR {DNA methylome} OR {RNA expression profile}))
#3	Gene Expression Regulatory (Mechanisms)	TITLE-ABS-KEY ({gene expression} OR ({gene expression} AND {profile pattern}) OR {gene regulation} OR {differentially expressed gene} OR {genetic transcription} OR {transcriptional regulation} OR {rna expression} OR {epigenetic} OR {differentially methylated region} OR {nucleosome positioning} OR {dna binding protein} OR {transcriptional factor} OR {micrornas}))
#4	Limits & Filters	#1 AND #2 AND #3

	Combined Concepts	(TITLE-ABS-KEY ({Sex Chromosome Aberration} OR (({sex chromosome} OR {x chromosome} OR {y chromosome}) AND ({aneuploid} OR {aberration} OR {abnormalities} OR {variation} OR {dosage} OR {alteration})) OR {turner syndrome} OR {X monosomy} OR {X0 karyotype} OR {klinefelter syndrome} OR {XXY} OR {XXYY} OR {XYY} OR {Triple X syndrome} OR {Triple X syndromes} OR {47 XXX} OR {extra X chromosome})) AND (TITLE-ABS-KEY ({gene expression} OR ({gene expression} AND {profile pattern}) OR {gene regulation} OR {differentially expressed gene} OR {genetic transcription} OR {transcriptional regulation} OR {rna expression} OR {epigenetic} OR {differentially methylated region} OR {nucleosome positioning} OR {dna binding protein} OR {transcriptional factor} OR {micrornas})) AND (TITLE-ABS-KEY ({gene expression profiling} OR {expression microarray} OR {high-throughput sequencing} OR {next generation sequencing} OR {deep sequencing} OR pyrosequencing OR {illumina sequencing} OR {ribosome profiling} OR {rna sequencing} OR {rna seq} OR rnaseq OR (transcriptome AND analysis) OR hictmap OR {chip seq} OR chipseq OR {chromatin immunoprecipitation sequencing} OR {atac seq} OR {transposase accessible chromatin} OR {single cell analysis} OR {single cell sequencing} OR {scrna seq} OR {scatac eq} OR {snrna seq} OR {bisulfite sequencing} OR wgbs OR {bs seq} OR {methylated dna immunoprecipitation} OR {medip seq} OR {methyl cpg binding domain sequencing} OR {mbd seq} OR {mre seq} OR {methylation array} OR {methylation microarray} OR hi-c OR {chromosome conformation capture} OR {chromatin conformation capture} OR {nucleosome mapping} OR {mnase seq} OR {faire seq} OR {dnase seq} OR hichip OR {merip seq} OR {m6a seq} OR transcriptome OR multiome OR {DNA methylation profile} OR {DNAm profile} OR {DNA methylome} OR {RNA expression profile})) AND (LIMIT-TO (DOCTYPE , "ar"))

Database: Web of Science (Core Collection)
Platform: Clarivate Analytics
Date Searched: 1/18/2024
Updated Search: 2/26/2025

	Concept:	Search Strategy:
#1	Sex Chromosomes	(TI=((('sex chromosom*' OR (('x chromosom*' OR 'y chromosom*') AND ('aneuploid*' OR 'aberration*' OR 'abnormalit*' OR 'variation*' OR 'dosage' OR 'alteration*')) OR 'turner syndrome*' OR (x NEAR/2 monosomy) OR (XO NEAR/2 karyotype) OR 'klinefelter syndrome*' OR 'xxy' OR 'xxyy' OR 'xyy' OR ('triple x' NEAR/2 syndrome*) OR ('47' NEAR/2 xxx) OR ('extra x' NEAR/4 chromosome*))) OR AB=((('sex chromosom*' OR (('x chromosom*' OR 'y chromosom*') AND ('aneuploid*' OR 'aberration*' OR 'abnormalit*' OR 'variation*' OR 'dosage' OR 'alteration*')) OR 'turner syndrome*' OR (x NEAR/2 monosomy) OR (XO NEAR/2 karyotype) OR 'klinefelter syndrome*' OR 'xxy' OR 'xxyy' OR 'xyy' OR ('triple x' NEAR/2 syndrome*) OR ('47' NEAR/2 xxx) OR ('extra x' NEAR/4 chromosome*))

#2	Gene Expression transcriptional regulation (Non-interventional Evaluation)	(TI=('gene expression profiling' OR (expression NEAR/3 microarray) OR (expression NEAR/3 microarray*) OR 'high-throughput sequencing' OR 'next generation sequenc*' OR 'deep sequencing' OR 'pyrosequencing' OR 'illumina sequencing' OR 'ribosome profiling' OR (rna NEAR/2 sequencing) OR (rna NEAR/2 seq) OR 'rnaseq' OR ('transcriptom*' AND 'analy*') OR 'hictmap' OR (chip NEAR/2 seq) OR 'chipseq' OR ('chromatin immunoprecipitation' NEAR/3 sequencing) OR (atac NEAR/3 seq) OR ('transposase accessible' NEAR/3 chromatin) OR ('single cell' NEAR/3 analysis) OR ('single cell' NEAR/3 sequencing) OR (scrna NEAR/2 seq) OR (scatac NEAR/2 seq) OR (snrna NEAR/2 seq) OR (bisulfite NEAR/3 sequencing) OR (bisulphite NEAR/3 sequencing) OR 'wgbs' OR (bs NEAR/2 seq) OR ('methylated dna' NEAR/3 immunoprecipitation) OR (medip NEAR/2 seq) OR ('methyl cpg binding domain' NEAR/4 'sequencing') OR (mbd NEAR/3 seq) OR (mre NEAR/2 seq) OR (methylation NEAR/2 array) OR (methylation NEAR/2 microarray) OR 'hi-c' OR (chromosome NEAR/3 'conformation capture') OR (chromatin NEAR/3 'conformation capture') OR (nucleosome NEAR/2 mapping) OR (mnase NEAR/3 seq) OR (faire NEAR/3 seq) OR (dnase NEAR/3 seq) OR 'hichip' OR (merip NEAR/2 seq) OR (m6a NEAR/2 seq) OR 'transcriptom*' OR 'multiom*' OR 'dna methylation profil*' OR 'dnam profil*' OR 'dna methylom*' OR 'rna expression profil*')) OR AB=('gene expression profiling' OR (expression NEAR/3 microarray) OR (expression NEAR/3 microarray*) OR 'high-throughput sequencing' OR 'next generation sequenc*' OR 'deep sequencing' OR 'pyrosequencing' OR 'illumina sequencing' OR 'ribosome profiling' OR (rna NEAR/2 sequencing) OR (rna NEAR/2 seq) OR 'rnaseq' OR ('transcriptom*' AND 'analy*') OR 'hictmap' OR (chip NEAR/2 seq) OR 'chipseq' OR ('chromatin immunoprecipitation' NEAR/3 sequencing) OR (atac NEAR/3 seq) OR ('transposase accessible' NEAR/3 chromatin) OR ('single cell' NEAR/3 analysis) OR ('single cell' NEAR/3 sequencing) OR (scrna NEAR/2 seq) OR (scatac NEAR/2 seq) OR (snrna NEAR/2 seq) OR (bisulfite NEAR/3 sequencing) OR (bisulphite NEAR/3 sequencing) OR 'wgbs' OR (bs NEAR/2 seq) OR ('methylated dna' NEAR/3 immunoprecipitation) OR (medip NEAR/2 seq) OR ('methyl cpg binding domain' NEAR/4 'sequencing') OR (mbd NEAR/3 seq) OR (mre NEAR/2 seq) OR (methylation NEAR/2 array) OR (methylation NEAR/2 microarray) OR 'hi-c' OR (chromosome NEAR/3 'conformation capture') OR (chromatin NEAR/3 'conformation capture') OR (nucleosome NEAR/2 mapping) OR (mnase NEAR/3 seq) OR (faire NEAR/3 seq) OR (dnase NEAR/3 seq) OR 'hichip' OR (merip NEAR/2 seq) OR (m6a NEAR/2 seq) OR 'transcriptom*' OR 'multiom*' OR 'dna methylation profil*' OR 'dnam profil*' OR 'dna methylom*' OR 'rna expression profil*')

#3	Gene Expression Regulatory (Mechanisms)	(TI=('gene expression' OR (('gene' AND 'expression') AND ('profile' OR 'pattern*')) OR (gene NEAR/2 expression) OR (gene NEAR/2 regulation) OR ((differentially NEAR/2 expressed) AND 'gene') OR (genetic NEAR/2 transcription) OR (transcriptional NEAR/2 regulation) OR (rna NEAR/2 expression) OR 'epigen*' OR (differentially NEAR/3 'methylated region*') OR (nucleosome NEAR/3 positioning) OR (dna NEAR/2 'binding protein*') OR (transcriptional NEAR/2 factor*) OR 'micrornas')) OR AB=('gene expression' OR (('gene' AND 'expression') AND ('profile' OR 'pattern*')) OR (gene NEAR/2 expression) OR (gene NEAR/2 regulation) OR ((differentially NEAR/2 expressed) AND 'gene') OR (genetic NEAR/2 transcription) OR (transcriptional NEAR/2 regulation) OR (rna NEAR/2 expression) OR 'epigen*' OR (differentially NEAR/3 'methylated region*') OR (nucleosome NEAR/3 positioning) OR (dna NEAR/2 'binding protein*') OR (transcriptional NEAR/2 factor*) OR 'micrornas')
#4	Limits & Filters	#3 AND #2 AND #1

Database: ProQuest Dissertations & Citation Theses Index
Platform: Clarivate Analytics
Date Searched: 1/18/2024
Updated Search: 2/26/2025

	Concept:	Search Strategy:
#1	Sex Chromosomes	(TI=((('sex chromosom*' OR 'x chromosom*' OR 'y chromosom*') AND ('aneuploid*' OR 'aberration*' OR 'abnormalit*' OR 'variation*' OR 'dosage' OR 'alteration*')) OR 'turner syndrome*' OR (x NEAR/2 monosomy) OR (XO NEAR/2 karyotype) OR 'klinefelter syndrome*' OR 'xxy' OR 'xxyy' OR 'xyy' OR ('triple x' NEAR/2 syndrome*) OR ('47' NEAR/2 xxx) OR ('extra x' NEAR/4 chromosome*))) OR AB=((('sex chromosom*' OR 'x chromosom*' OR 'y chromosom*') AND ('aneuploid*' OR 'aberration*' OR 'abnormalit' OR 'variation*' OR 'dosage' OR 'alteration*')) OR 'turner syndrome*' OR (x NEAR/2 monosomy) OR (XO NEAR/2 karyotype) OR 'klinefelter syndrome*' OR 'xxy' OR 'xxyy' OR 'xyy' OR ('triple x' NEAR/2 syndrome*) OR ('47' NEAR/2 xxx) OR ('extra x' NEAR/4 chromosome*))

#2	Gene Expression transcriptional regulation (Non-interventional Evaluation)	(TI=('gene expression profiling' OR (expression NEAR/3 microarray) OR (expression NEAR/3 microarray*) OR 'high-throughput sequencing' OR 'next generation sequenc*' OR 'deep sequencing' OR 'pyrosequencing' OR 'illumina sequencing' OR 'ribosome profiling' OR (rna NEAR/2 sequencing) OR (rna NEAR/2 seq) OR 'rnaseq' OR ('transcriptom*' AND 'analy*') OR 'hictmap' OR (chip NEAR/2 seq) OR 'chipseq' OR ('chromatin immunoprecipitation' NEAR/3 sequencing) OR (atac NEAR/3 seq) OR ('transposase accessible' NEAR/3 chromatin) OR ('single cell' NEAR/3 analysis) OR ('single cell' NEAR/3 sequencing) OR (scrna NEAR/2 seq) OR (scatac NEAR/2 seq) OR (snrna NEAR/2 seq) OR (bisulfite NEAR/3 sequencing) OR (bisulphite NEAR/3 sequencing) OR 'wgbs' OR (bs NEAR/2 seq) OR ('methylated dna' NEAR/3 immunoprecipitation) OR (medip NEAR/2 seq) OR ('methyl cpg binding domain' NEAR/4 'sequencing') OR (mbd NEAR/3 seq) OR (mre NEAR/2 seq) OR (methylation NEAR/2 array) OR (methylation NEAR/2 microarray) OR 'hi-c' OR (chromosome NEAR/3 'conformation capture') OR (chromatin NEAR/3 'conformation capture') OR (nucleosome NEAR/2 mapping) OR (mnase NEAR/3 seq) OR (faire NEAR/3 seq) OR (dnase NEAR/3 seq) OR 'hichip' OR (merip NEAR/2 seq) OR (m6a NEAR/2 seq) OR 'transcriptom*' OR 'multiom*' OR 'dna methylation profil*' OR 'dnam profil*' OR 'dna methylom*' OR 'rna expression profil*')) OR AB=('gene expression profiling' OR (expression NEAR/3 microarray) OR (expression NEAR/3 microarray*) OR 'high-throughput sequencing' OR 'next generation sequenc*' OR 'deep sequencing' OR 'pyrosequencing' OR 'illumina sequencing' OR 'ribosome profiling' OR (rna NEAR/2 sequencing) OR (rna NEAR/2 seq) OR 'rnaseq' OR ('transcriptom*' AND 'analy*') OR 'hictmap' OR (chip NEAR/2 seq) OR 'chipseq' OR ('chromatin immunoprecipitation' NEAR/3 sequencing) OR (atac NEAR/3 seq) OR ('transposase accessible' NEAR/3 chromatin) OR ('single cell' NEAR/3 analysis) OR ('single cell' NEAR/3 sequencing) OR (scrna NEAR/2 seq) OR (scatac NEAR/2 seq) OR (snrna NEAR/2 seq) OR (bisulfite NEAR/3 sequencing) OR (bisulphite NEAR/3 sequencing) OR 'wgbs' OR (bs NEAR/2 seq) OR ('methylated dna' NEAR/3 immunoprecipitation) OR (medip NEAR/2 seq) OR ('methyl cpg binding domain' NEAR/4 'sequencing') OR (mbd NEAR/3 seq) OR (mre NEAR/2 seq) OR (methylation NEAR/2 array) OR (methylation NEAR/2 microarray) OR 'hi-c' OR (chromosome NEAR/3 'conformation capture') OR (chromatin NEAR/3 'conformation capture') OR (nucleosome NEAR/2 mapping) OR (mnase NEAR/3 seq) OR (faire NEAR/3 seq) OR (dnase NEAR/3 seq) OR 'hichip' OR (merip NEAR/2 seq) OR (m6a NEAR/2 seq) OR 'transcriptom*' OR 'multiom*' OR 'dna methylation profil*' OR 'dnam profil*' OR 'dna methylom*' OR 'rna expression profil*')

#3	Gene Expression Regulatory (Mechanisms)	(TI=('gene expression' OR (('gene' AND 'expression') AND ('profile' OR 'pattern*')) OR (gene NEAR/2 expression) OR (gene NEAR/2 regulation) OR ((differentially NEAR/2 expressed) AND 'gene') OR (genetic NEAR/2 transcription) OR (transcriptional NEAR/2 regulation) OR (rna NEAR/2 expression) OR 'epigen*' OR (differentially NEAR/3 'methylated region*') OR (nucleosome NEAR/3 positioning) OR (dna NEAR/2 'binding protein*') OR (transcriptional NEAR/2 factor*) OR 'micrornas')) OR AB=('gene expression' OR (('gene' AND 'expression') AND ('profile' OR 'pattern*')) OR (gene NEAR/2 expression) OR (gene NEAR/2 regulation) OR ((differentially NEAR/2 expressed) AND 'gene') OR (genetic NEAR/2 transcription) OR (transcriptional NEAR/2 regulation) OR (rna NEAR/2 expression) OR 'epigen*' OR (differentially NEAR/3 'methylated region*') OR (nucleosome NEAR/3 positioning) OR (dna NEAR/2 'binding protein*') OR (transcriptional NEAR/2 factor*) OR 'micrornas')
#4	Limits & Filters	#3 AND #2 AND #1

Database: Preprints Citation Index
Platform: Clarivate Analytics
Date Searched: 1/18/2024
Updated Search: 2/26/2025

	Concept:	Search Strategy:
#1	Sex Chromosomes	(TI=((('sex chromosom*' OR 'x chromosom*' OR 'y chromosom*') AND ('aneuploid*' OR 'aberration*' OR 'abnormalit*' OR 'variation*' OR 'dosage' OR 'alteration*')) OR 'turner syndrome*' OR (x NEAR/2 monosomy) OR (XO NEAR/2 karyotype) OR 'klinefelter syndrome*' OR 'xxy' OR 'xxyy' OR 'xyy' OR ('triple x' NEAR/2 syndrome*) OR ('47' NEAR/2 xxx) OR ('extra x' NEAR/4 chromosome*))) OR AB=((('sex chromosom*' OR 'x chromosom*' OR 'y chromosom*') AND ('aneuploid*' OR 'aberration*' OR 'abnormalit' OR 'variation*' OR 'dosage' OR 'alteration*')) OR 'turner syndrome*' OR (x NEAR/2 monosomy) OR (XO NEAR/2 karyotype) OR 'klinefelter syndrome*' OR 'xxy' OR 'xxyy' OR 'xyy' OR ('triple x' NEAR/2 syndrome*) OR ('47' NEAR/2 xxx) OR ('extra x' NEAR/4 chromosome*))

#2	Gene Expression transcriptional regulation (Non-interventional Evaluation)	(TI=('gene expression profiling' OR (expression NEAR/3 microarray) OR (expression NEAR/3 microarray*) OR 'high-throughput sequencing' OR 'next generation sequenc*' OR 'deep sequencing' OR 'pyrosequencing' OR 'illumina sequencing' OR 'ribosome profiling' OR (rna NEAR/2 sequencing) OR (rna NEAR/2 seq) OR 'rnaseq' OR ('transcriptom*' AND 'analy*') OR 'hictmap' OR (chip NEAR/2 seq) OR 'chipseq' OR ('chromatin immunoprecipitation' NEAR/3 sequencing) OR (atac NEAR/3 seq) OR ('transposase accessible' NEAR/3 chromatin) OR ('single cell' NEAR/3 analysis) OR ('single cell' NEAR/3 sequencing) OR (scrna NEAR/2 seq) OR (scatac NEAR/2 seq) OR (snrna NEAR/2 seq) OR (bisulfite NEAR/3 sequencing) OR (bisulphite NEAR/3 sequencing) OR 'wgbs' OR (bs NEAR/2 seq) OR ('methylated dna' NEAR/3 immunoprecipitation) OR (medip NEAR/2 seq) OR ('methyl cpg binding domain' NEAR/4 'sequencing') OR (mbd NEAR/3 seq) OR (mre NEAR/2 seq) OR (methylation NEAR/2 array) OR (methylation NEAR/2 microarray) OR 'hi-c' OR (chromosome NEAR/3 'conformation capture') OR (chromatin NEAR/3 'conformation capture') OR (nucleosome NEAR/2 mapping) OR (mnase NEAR/3 seq) OR (faire NEAR/3 seq) OR (dnase NEAR/3 seq) OR 'hichip' OR (merip NEAR/2 seq) OR (m6a NEAR/2 seq) OR 'transcriptom*' OR 'multiom*' OR 'dna methylation profil*' OR 'dnam profil*' OR 'dna methylom*' OR 'rna expression profil*')) OR AB=('gene expression profiling' OR (expression NEAR/3 microarray) OR (expression NEAR/3 microarray*) OR 'high-throughput sequencing' OR 'next generation sequenc*' OR 'deep sequencing' OR 'pyrosequencing' OR 'illumina sequencing' OR 'ribosome profiling' OR (rna NEAR/2 sequencing) OR (rna NEAR/2 seq) OR 'rnaseq' OR ('transcriptom*' AND 'analy*') OR 'hictmap' OR (chip NEAR/2 seq) OR 'chipseq' OR ('chromatin immunoprecipitation' NEAR/3 sequencing) OR (atac NEAR/3 seq) OR ('transposase accessible' NEAR/3 chromatin) OR ('single cell' NEAR/3 analysis) OR ('single cell' NEAR/3 sequencing) OR (scrna NEAR/2 seq) OR (scatac NEAR/2 seq) OR (snrna NEAR/2 seq) OR (bisulfite NEAR/3 sequencing) OR (bisulphite NEAR/3 sequencing) OR 'wgbs' OR (bs NEAR/2 seq) OR ('methylated dna' NEAR/3 immunoprecipitation) OR (medip NEAR/2 seq) OR ('methyl cpg binding domain' NEAR/4 'sequencing') OR (mbd NEAR/3 seq) OR (mre NEAR/2 seq) OR (methylation NEAR/2 array) OR (methylation NEAR/2 microarray) OR 'hi-c' OR (chromosome NEAR/3 'conformation capture') OR (chromatin NEAR/3 'conformation capture') OR (nucleosome NEAR/2 mapping) OR (mnase NEAR/3 seq) OR (faire NEAR/3 seq) OR (dnase NEAR/3 seq) OR 'hichip' OR (merip NEAR/2 seq) OR (m6a NEAR/2 seq) OR 'transcriptom*' OR 'multiom*' OR 'dna methylation profil*' OR 'dnam profil*' OR 'dna methylom*' OR 'rna expression profil*')

#3	Gene Expression Regulatory (Mechanisms)	(TI=('gene expression' OR (('gene' AND 'expression') AND ('profile' OR 'pattern*')) OR (gene NEAR/2 expression) OR (gene NEAR/2 regulation) OR ((differentially NEAR/2 expressed) AND 'gene') OR (genetic NEAR/2 transcription) OR (transcriptional NEAR/2 regulation) OR (rna NEAR/2 expression) OR 'epigen*' OR (differentially NEAR/3 'methylated region*') OR (nucleosome NEAR/3 positioning) OR (dna NEAR/2 'binding protein*') OR (transcriptional NEAR/2 factor*) OR 'micrornas')) OR AB=('gene expression' OR (('gene' AND 'expression') AND ('profile' OR 'pattern*')) OR (gene NEAR/2 expression) OR (gene NEAR/2 regulation) OR ((differentially NEAR/2 expressed) AND 'gene') OR (genetic NEAR/2 transcription) OR (transcriptional NEAR/2 regulation) OR (rna NEAR/2 expression) OR 'epigen*' OR (differentially NEAR/3 'methylated region*') OR (nucleosome NEAR/3 positioning) OR (dna NEAR/2 'binding protein*') OR (transcriptional NEAR/2 factor*) OR 'micrornas')
#4	Limits & Filters	#3 AND #2 AND #1

Database: bioRxiv (Preprints)
Platform: Cold Spring Harbor
Date Searched: 1/18/2024
Updated Search: 2/26/2025

	Concept:	Search Strategy:
		abstract or title ""sex chromosome*" AND "gene expression" AND (regulation* OR transcription* OR pattern*)" (match phrase words)

Database: medrxiv (Preprints)
Platform: Cold Spring Harbor
Date Searched: 1/18/2024
Updated Search: 2/26/2025

	Concept:	Search Strategy:
		abstract or title ""sex chromosome*" AND "gene expression" AND (regulation* OR transcription* OR pattern*)" (match phrase words)

Website: ClinicalTrials.gov
Date Searched: 1/18/2024
Updated Search: 2/26/2025
Limits: Recruiting/Not yet recruiting

	Concept:	Search Strategy:
		(Sex chromosome AND Gene Profile, Gene Signature; Gene Expression Profiling)
