# Supplementary material for: Sex chromosome aneuploidy impacts on human gene expression and regulation: a systematic review
Source: Mol Med. 2025 Dec 30;32:11. doi: 10.1186/s10020-025-01404-1 (PMC12859965; doi:10.1186/s10020-025-01404-1)
Supplement: Supplementary file 7 — Supplementary Material 7. Table S2. Inclusion and exclusion criteria. [file 10020_2025_1404_MOESM7_ESM.pdf]

Table S1. Inclusion and exclusion criteria, based on the Population, Exposure, Comparator, and Outcomes (PECO) framework.

	Inclusion	Exclusion
Population	Individuals with sex chromosome aneuploidies.	Individuals with mosaic sex chromosome aneuploidies.
		Individuals with partial sex chromosome deletions (e.g. 46,XYq- karyotype) or with sex chromosome microdeletions.
		Sex chromosome aneuploidy in cells or tissues of somatic origin, such as in cancer.
		Aneuploid cells or tissues that involve autosomal chromosomes, such as polyploid or non-diploid cells.
		Use of animal or plants models only.
		X chromosome-related disorders (i.e. Fragile X syndrome)
Intervention / Exposure	Techniques, assays, or methods designed for unbiased or genome-wide measurement of gene expression or mechanisms that regulate transcription, such as gene DNA methylation, chromatin structure, or other epigenetic components.	Techniques, assays or methods that only evaluate single nucleotide polymorphisms or copy number variations (such as whole genome sequencing, exome sequencing or comparative genomic hybridization)
		Studies focused solely on a single gene or pre-defined subsets of genes. Some examples of techniques designed for specific genes are Polymerase chain reaction (PCR), Real time PCR (RT-PCR) or quantitative RT-PCR (qRT-PCR), Northern Blot, Southern blot, Western Blot, Gene-specific methylation.
Comparator	Patients with no sex chromosome aneuploidy.	Individuals with mosaic sex chromosome aneuploidies
	Patients with a different sex chromosome aneuploidy.	Individuals with other population exclusion criteria
Outcome	Changes on genome structure and function, reported at the cellular and/or molecular level.	Different outcome measures (not directly related to genome structure or function), such as prenatal screening, specific treatments, or any other unrelated diagnostic or therapeutic measure.
