# Supplementary material for: Sex chromosome aneuploidy impacts on human gene expression and regulation: a systematic review
Source: Mol Med. 2025 Dec 30;32:11. doi: 10.1186/s10020-025-01404-1 (PMC12859965; doi:10.1186/s10020-025-01404-1)
Supplement: Supplementary file 10 — Supplementary Material 10. Figures S1-S7. [file 10020_2025_1404_MOESM10_ESM.pdf]

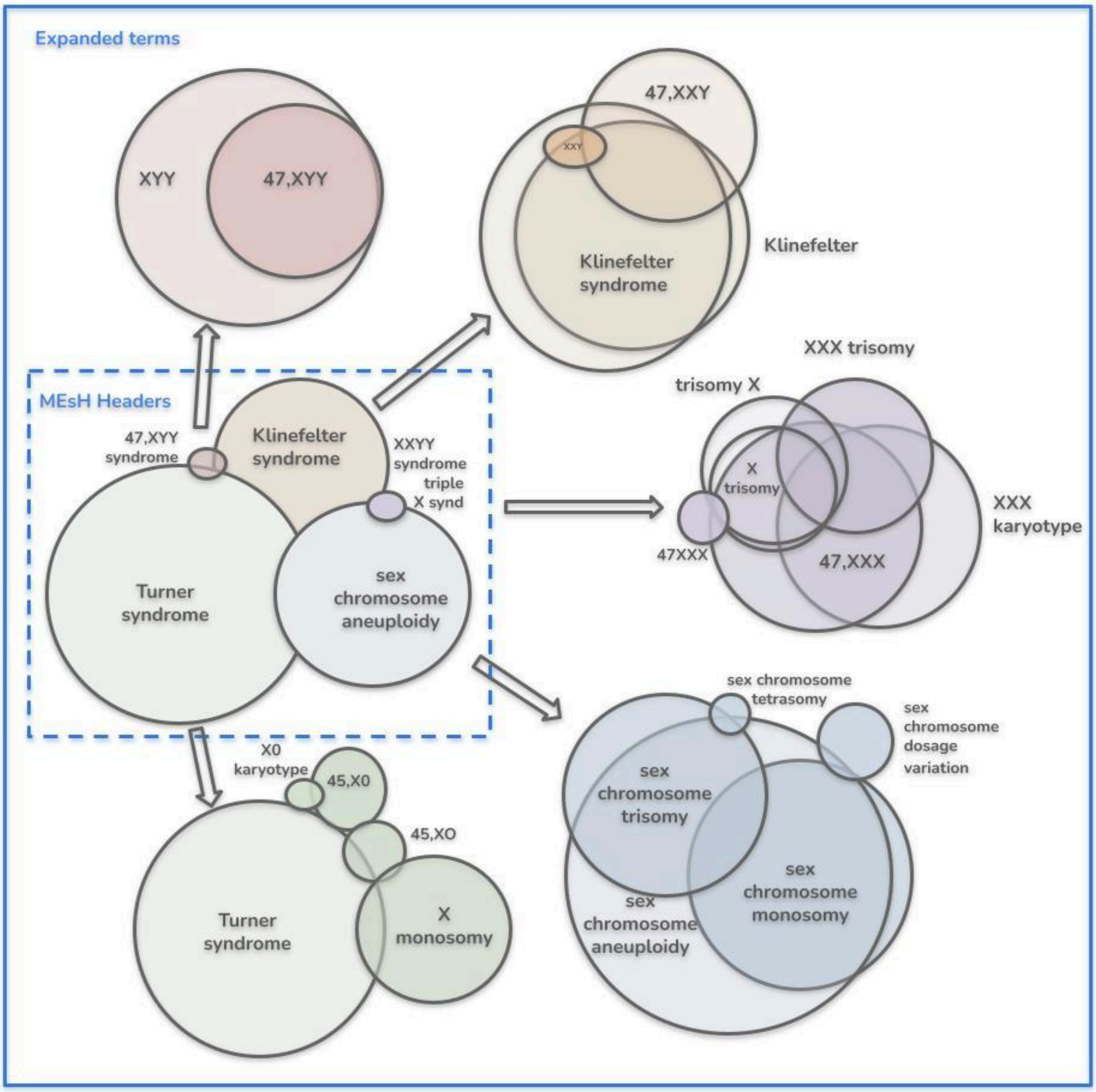

Supplementary Figure 1.Graphical representation of MESH and semantically related terms. Visualization was performed with the goal of optimizing the combination of search terms during the search building. The figure depicts the semantic space of ‘Sex Chromosome Aneuploidy’ and related terms, the same process was performed for other search aspects (techniques and genomic elements studied). The visualization was made with the tool PubVenn (Sperr E. PubVenn [Internet]. 2015 [updated search in february 2025]. Available from https://pubvenn.appspot.com/).

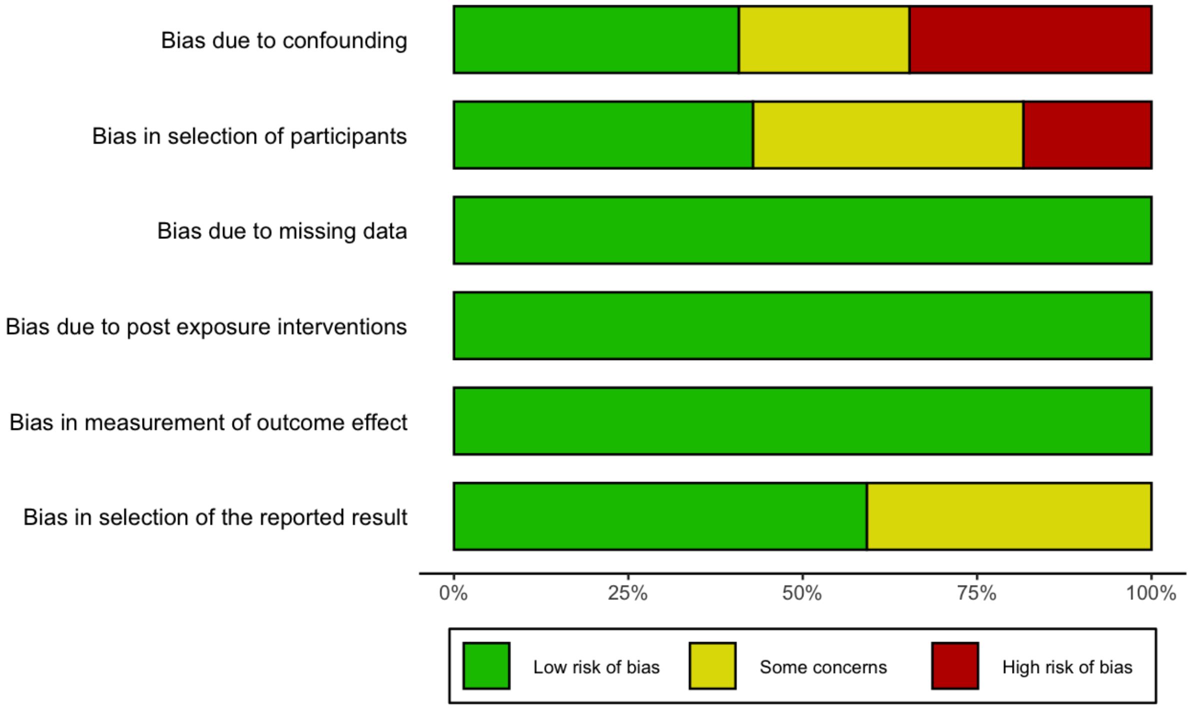

Supplementary Figure 2. Risk of bias assessment summary. Each bar represents one domain colored by the aggregated evaluation of all studies in it. Most affected domains are confounding and selection of participants.

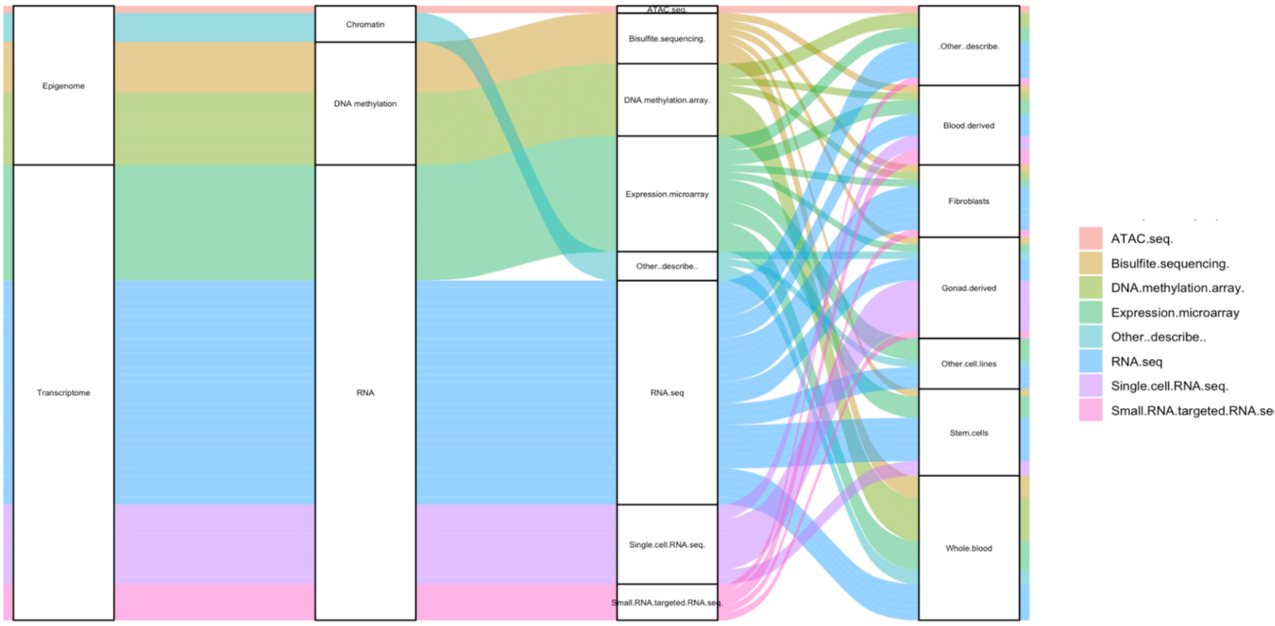

Supplementary Figure 3. Tests tissues and techniques performed across studies. Representation of link between different methods performed over different tissues and different genomic components.

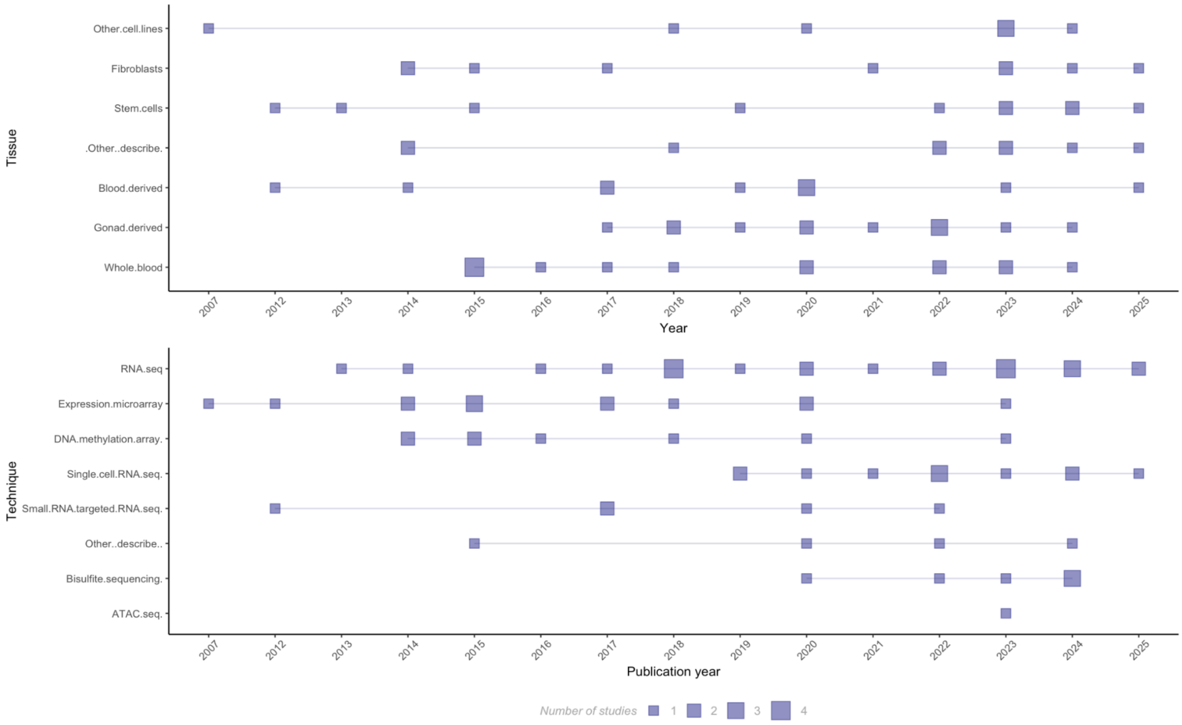

Supplementary Figure 4. SCAs tissues and techniques studied over time. Representation of tissues or cell type (top) and evaluation methods utilized (bottom) on the studies on sex chromosome aneuploidies over the years.

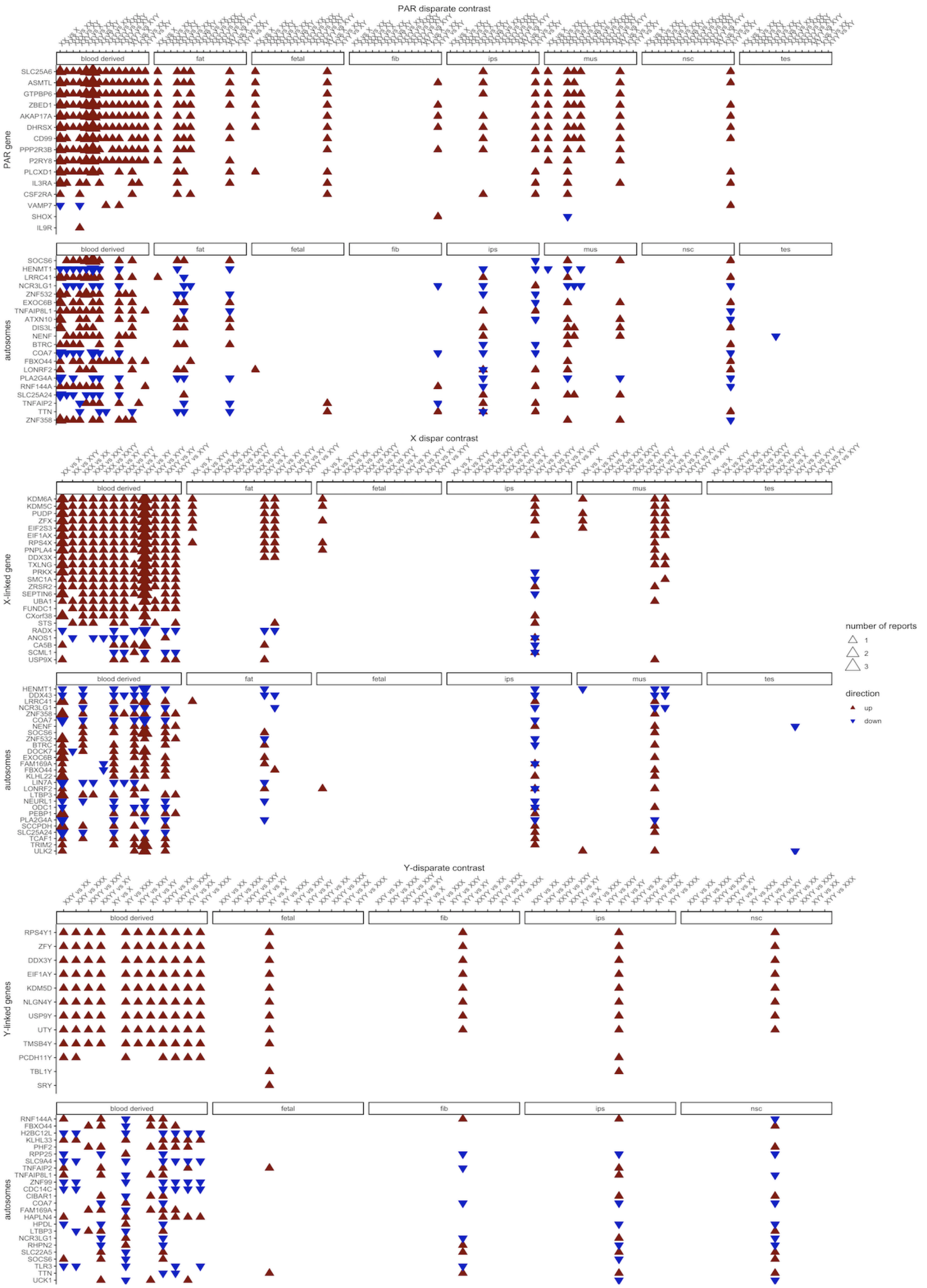

PAR disparate contrast
blood derived
fat
fetal
fib
ips
mus
nsc
tes
PAR gene
SLC25A6
ASMTL
GTPBP6
ZBED1
AKAP17A
DHRSX
CD99
PPP2R3B
P2RY8
PLCXD1
IL3RA
CSF2RA
VAMP7
SHOX
IL9R
autosomes
SOCS6
HENMT1
LRRC41
NCR3LG1
ZNF532
EXOC6B
TNFAIP8L1
ATXN10
DIS3L
NENF
BTRC
COA7
FBXO44
LONRF2
PLA2G4A
RNF144A
SLC25A24
TNFAIP2
TTN
ZNF358
X dispar contrast
X-linked gene
KDM6A
KDM5C
PUDP
ZFX
EIF2S3
EIF1AX
RPS4X
PNPLA4
DDX3X
TXLNG
PRKX
SMC1A
ZRSR2
SEPTIN6
UBA1
FUNDC1
CXorf38
STS
RADX
ANOS1
CA5B
SCML1
USP9X
HENMT1
DDX43
LRRC41
NCR3LG1
ZNF358
COA7
NENF
SOCS6
ZNF532
BTRC
DOCK7
EXOC6B
FAM169A
FBXO44
KLHL22
LIN7A
LONRF2
LTBP3
NEURL1
ODC1
PEBP1
PLA2G4A
SCCPDH
SLC25A24
TCAF1
TRIM2
ULK2
number of reports
1
2
3
direction
up
down
Y-disparate contrast
Y-linked genes
RPS4Y1
ZFY
DDX3Y
EIF1AY
KDM5D
NLGN4Y
USP9Y
UTY
TMSB4Y
PCDH11Y
TBL1Y
SRY
RNF144A
FBXO44
H2BC12L
KLHL33
PHF2
RPP25
SLC9A4
TNFAIP2
TNFAIP8L1
ZNF99
CDC14C
CIBAR1
COA7
FAM169A
HAPLN4
HPDL
LTBP3
NCR3LG1
RHPN2
SLC22A5
SOCS6
TLR3
TTN
UCK1

Supplementary Figure 5. Recurrently reported DEGs in gene expression studies of SCAs by reported tissue. Dot plots showing the direction of differential expression for each gene in the informative contrasts (arrow direction and color), the number of reports of differential expression (arrow size) on each studied contrasts. Blank spaces are indicative of contrasts not studied in that tissue or DEG not reported. Blood-derived and fetal tissues have been collapsed for visualization, itemized information is available in Table S3.

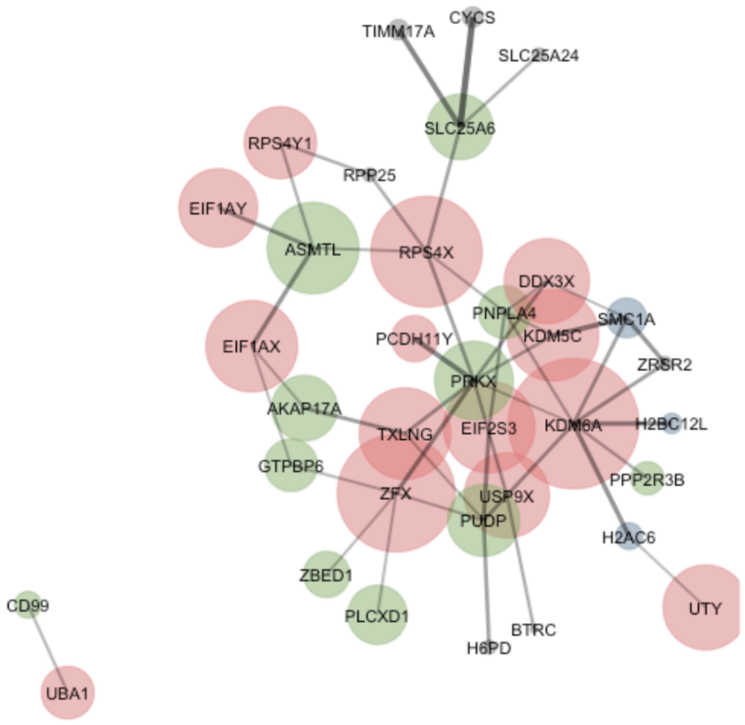

Supplementary Figure 6. Intercluster connections between the three main clusters of frequent DEGs in SCAs. Edge width represents confidence score, node size represents degree, and color represents cluster membership.

Study ID*	Source(s) used (doi)	Number of eligible participants **	(Contrasts evaluated)	Result available for inclusion in Meta-analysis 1 (Whole Blood)	Result available for inclusion in Meta-analysis 2 (Blood-derived)	Result available for inclusion in Meta-analysis 4 (Fibroblasts)	Result available for inclusion in Meta-analysis 3 (Gonad-derived)	Result available for inclusion in Meta-analysis 5 (Stem cells)	Is Meta-analysis feasible on this same tissue?
Nielsen 2020	10.1002/ajmg.c.31799	7 | 7		X					
Skakkebæk 2018	10.1038/s41598-018-31780-0	67		X					NO
Trolle 2016	10.1038/srep34220	35		X					
Viuff 2023	10.1186/s13073-023-01169-4	36 | 62		✓					
Zhang 2020	10.1073/pnas.1910003117	13-14			✓				
Cimino 2017	merged	10			?				NO
Liu 2023	10.1073/pnas.2218478120	32 | 46 | 30 | 31			✓	✓			
Biradar 2021	10.1007/s11626-021-00604-3	1				***			
Rajpathak 2014	10.1371/journal.pone.0100076	1				***			NO
SanRoman 2023	10.1016/j.xgen.2023.100259	31 | 9 | 38 | 13 | 1 | 4 | 3 | 13 | 2				?			
Willems 2022	10.1038/s41598-022-26011-6	5					✓		
Winge 2018 (a)	0.1093/hmg/ddx411	2					***		NO
Winge 2018 (b)	10.1038/s41419-018-0671-1	3					✓		
Astro 2023	https://doi.org/10.1530/EC-22-0515	4 | 5						✓	
Astro 2022	10.3389/fcell.2021.801597	2 | 2						✓	NO (iPSCs)
Panula 2019	10.1093/humrep/dez134	2						***	
Zhang 2013	10.1186/1471-2164-14-S5-S8	2						***	
Licciardi	10.1038/s41598-018-33279-0	1 | 1						? ***	NO (HeSCs)

Legend

(green)	Low reporting bias
(yellow)	Moderate reporting bias
(red)	High reporting bias
***	Sample size ≤ 3
✓	Complete gene list w/ pval & FC
~	Complete gene list lacking pval | FC
X	Selective reporting
?	Unclear / Inconsistency / No list

Supplementary Figure 7. Evaluation of missing evidence for meta-analysis, adapted from "“Tool for assessing Risk of bias due to missing evidence in a synthesis”. Scarce studies using the same tissue and contrast, or low sample sample size or inconsistent reporting prevented tissue-specific analyses.
